# Correlation of cognitive dysfunctions and diffusion tensor MRI measures in subjects with RRMS

**DOI:** 10.3389/fnagi.2025.1661821

**Published:** 2025-11-13

**Authors:** Alessio Mirabile, Carla Susinna, Giovanni Luca Cipriano, Giangaetano D’Aleo, Carmela Rifici, Edoardo Sessa, Angelo Quartarone, Viviana Lo Buono

**Affiliations:** IRCCS Centro Neurolesi "Bonino-Pulejo", Messina, Italy

**Keywords:** multiple sclerosis, relapsing-remitting multiple sclerosis, diffusion tensor imaging, cognition, neuropsychology, white matter

## Abstract

**Background:**

Cognitive dysfunction is a common impairment observed in individuals with Multiple Sclerosis (MS), affecting key domains such as attention, information processing speed, memory, and executive functions. This deficit is typically identified through comprehensive neuropsychological assessments, which represent the gold standard for cognitive evaluation. In recent years, increasing efforts have been made to integrate neuropsychological findings with advanced neuroimaging techniques to better understand the neural substrates of cognitive dysfunction. Among these, Diffusion Tensor Imaging (DTI) has emerged as a valuable tool for investigating microstructural changes in white matter (WM) that may underlie cognitive deficits in MS. However, despite its clinical utility, the pathophysiological mechanisms contributing to cognitive impairment, particularly in subjects with Relapsing–Remitting MS (RRMS), remain complex and not yet fully understood. This review focuses on studies investigating WM alterations measured by DTI and their correlation with cognitive dysfunction as assessed through neuropsychological testing.

**Method:**

Papers were identified by searching in PubMed, Embase, Web of Science and Scopus databases from 2002 - the years, of first related published article, − to December 2024. From the initial 853, we included only 34 studies that met to eligibility criteria.

**Results:**

In subjects with RRMS, WM alterations, assessed through DTI, were found to correlate with cognitive dysfunction, as measured by standardized neuropsychological tests. These alterations were observed both in global WM and in specific regions, including the corpus callosum, thalamus, hippocampus, cerebellar structure, cingulum, and cerebral fascicles.

**Conclusion:**

These findings underscore the relevance of integrating neuropsychological assessment with advanced neuroimaging techniques, such as DTI, to enhance our understanding of cognitive impairment in RRMS. DTI-derived measures of WM integrity show promise as potential biomarkers of cognitive dysfunction, while cognitive profiling can help localize underlying neuropsychological damage. This integrated approach may improve early detection of cognitive alterations and support the development of targeted therapeutic strategies aimed at preserving cognitive functioning in individuals with MS.

**Systematic review registration:**

https://www.crd.york.ac.uk/PROSPERO/view/CRD420251073195, Identifier CRD420251073195.

## 1 Introduction

Multiple sclerosis (MS) is an autoimmune, inflammatory, neurodegenerative disease that damages the brain and spinal cord by causing demyelination. It is marked by neurological symptoms and signs which contribute to physical disability (motor, sensory, and visual deficits, etc.), cognitive dysfunction, and fatigue even in the early stages of the disease. According to an estimate from the MS International Federation of Atlas, approximately 2.8 million people worldwide live with a diagnosis of MS. Globally, MS prevalence is 35.9 cases per 100,000 residents, with a higher incidence in women (Walton et al., 2020). Cognitive dysfunction is a major contributor to MS-related disability, with prevalence estimates ranging from 40 to 65% during the course of the disease (Chiaravalloti and DeLuca, 2008). Its frequency and severity may increase over time, becoming more evident in cases of MS progressive form. The presence of cognitive dysfunction is associated with a worse prognosis, reduced social participation, and lower quality of life (Rao et al., 1991; Amato et al., 2006). The pathophysiological mechanisms underlying cognitive dysfunction in MS have a multifactorial etiology, e.g., alterations in nerve conduction due to demyelination processes; axonal damage within cognitive networks; failure of compensatory mechanisms due to brain injury (Prins et al., 2015). Magnetic resonance imaging (MRI) studies have consistently highlighted correlations between the progression of cognitive dysfunction in MS and increased tissue damage, brain atrophy, and alterations in functional connectivity, particularly within the prefrontal cortex and limbic system (Zhang et al., 2021; Keser et al., 2018).

Among advanced MRI modalities, Diffusion Tensor Imaging (DTI) has emerged as a valuable tool for investigating organization and microstructural brain alterations that underlie cognitive dysfunction in MS. DTI allows for the *in vivo* assessment of white matter (WM) integrity by quantifying the diffusion properties of water molecules within neural tissues (Lope-Piedrafita, 2018). This technique is particularly sensitive to subtle tissue damage that may not be detectable with conventional MRI, including abnormalities in both WM lesions and apparently normal-appearing brain tissue (NABT), as well as in cortical and deep grey matter regions (Hori et al., 2022; Kolasa et al., 2019).

Key DTI-derived metrics include fractional anisotropy (FA), which reflects the directionality of water diffusion and serves as an indicator of WM fiber integrity (Beaulieu, 2002); radial diffusivity (RD), which represents the average diffusion of water molecules perpendicular to the principal fiber orientation and is particularly sensitive to myelin integrity (Song et al., 2002); axial diffusivity (AD), reflecting water diffusion parallel to the main fiber direction and often associated with axonal integrity (Song et al., 2003); mean diffusivity (MD), reflecting the overall magnitude of water diffusion and indicative of tissue density and extracellular space (Alexander et al., 2007); and the apparent diffusion coefficient (ADC), which quantifies the ease of water molecule movement within tissue and is often increased in areas of microstructural damage (Bihan et al., 2001).

Alterations in these parameters have been associated with demyelination, axonal loss, and disruption of cellular membranes, thereby providing insights into the microstructural substrates of cognitive dysfunction in MS (Andersen et al., 2018; Sijens et al., 2006). Importantly, abnormalities detected in NABT through DTI may precede the formation of new lesions or occur concurrently in homologous contralateral regions, suggesting a diffuse and dynamic pathological process (Bao et al., 2022).

Rao et al. (1989) suggest that cognitive dysfunction is caused by a disruption of cortico-subcortical circuits, which connect the frontal cortices to the thalamus and basal ganglia. However, other studies have reported that posterior brain areas and the corpus callosum (CC) may also play a role in cognitive impairment (Mesaros et al., 2009; Granberg et al., 2015). Furthermore, it has been suggested that a reduction in information processing speed may be associated with difficulties in sensory and motor functions (Dineen et al., 2009; Roosendaal et al., 2009).

Although the so-called “clinical–radiological paradox” describes the imperfect correlation between radiological findings and clinical symptoms (Rocca et al., 2015; Sumowski and Leavitt, 2013), cognitive dysfunction is frequently observed in MS subjects, affecting multiple domains such as attention, information processing speed (IPS), memory, and executive control (De Meo et al., 2021; Lugosi et al., 2024). The Symbol Digit Modality Test (SDMT) and the Paced Auditory Serial Addition Test (PASAT) are among the most used tests for evaluating cognitive deficits associated with lesions in specific brain areas in MS, measuring attention, concentration, and information processing speed (Matias-Guiu et al., 2018; Berrigan et al., 2022). Despite its clinical relevance, the underlying mechanisms of cognitive dysfunction in MS remain complex and not fully understood.

This review was conducted to clarify the current state of knowledge regarding the correlation between WM damage, as detected through DTI, and cognitive dysfunction assessed by neuropsychological evaluation.

## 2 Materials and method

### 2.1 Search strategy

A protocol for this systematic review was registered in PROSPERO (2025 ID: CRD420251073195). The review was conducted in accordance with the PRISMA (Preferred Reporting Items for Systematic Reviews and Meta-Analyses) guidelines. The PRISMA flow diagram was employed to provide a visual overview of the study selection process, encompassing the stages of identification, screening, eligibility, and inclusion. The PRISMA guidelines were followed to identify and analyse DTI studies investigating WM and cognitive function in MS patients. This systematic review did not involve human nor animal data collection.

Papers were identified (from September 2024 to December 2024) by searching in PubMed, Embase, Web of Science and Scopus database from 2002 - the years, of first related published article, − to December 2024. The search combined the following terms (“multiple sclerosis”[MeSH Terms] OR (“multiple”[All Fields] AND “sclerosis”[All Fields]) OR “multiple sclerosis”[All Fields] OR (“sclerosis”[All Fields] AND “multiple”[All Fields]) OR “sclerosis multiple”[All Fields]) AND (“diffusion tensor imaging”[MeSH Terms] OR (“diffusion”[All Fields] AND “tensor”[All Fields] AND “imaging”[All Fields]) OR “diffusion tensor imaging”[All Fields]) AND (“cognition”[MeSH Terms] OR “cognition”[All Fields] OR “cognitions”[All Fields] OR “cognitive”[All Fields] OR “cognitively”[All Fields] OR “cognitives”[All Fields]).

### 2.2 Data extraction

Two independent reviewers (AM and CS) screened titles and abstracts and extracted relevant data from the included studies. Extracted information included the authorship, year of publication, country of origin, study design, sample size, sex proportion, age, Expanded Disability Status Scale (EDSS) scores, disease duration, MRI device and strength, MRI acquisition sequence, MRI analysis software, cognitive test scores, and the main findings regarding the correlation between DTI measurements and neuropsychological evaluation in people with MS. The search results were imported into Excel software, where the two researchers independently removed duplicates and screened titles and abstracts based on predefined eligibility criteria. Studies that utilized DTI modalities in MS and assessed cognitive domains using standardized neuropsychological measures were identified and subjected to a comprehensive full-text review. We consider standardized neuropsychological instruments, validated tests that reliably assess cognitive domains, allowing for comparisons across participants and studies. In this context, cognitive performance refers to individuals’ abilities across different cognitive domains, such as memory, attention, processing speed, and executive functions. The agreement between the two independent reviewers was evaluated using Cohen’s kappa statistic. A kappa value greater than 0.61 was considered indicative of substantial agreement, ensuring a robust assessment of inter-rater reliability during the data extraction process.

### 2.3 Study selection

These researchers read the full-text articles deemed suitable for the study and performed data collection to reduce the risk of bias. Any disagreements among the researchers were resolved by consulting a senior reviewer (VLB).

Studies were included after they fulfilled the following criteria: (a) use of DTI; (b) studies assessed the relationship between WM damage and cognitive functions (c); study with only RRMS subjects (d) studies using standardized neuropsychological measures (e); presence of a control group (f); observational design, including case–control, cross–sectional, and cohort studies (either retrospective or prospective); (g) original articles written in English.

The exclusion criteria were: (a) reviews, single case studies, conference abstracts, protocols, editorials, letters, book chapters, case reports, and case series (b); pharmacological studies (c); paediatric onset study (d); studies with methodological ambiguities o insufficiently described methods (e); studies with incomplete, insufficient, or missing data.

**Figure 1:**
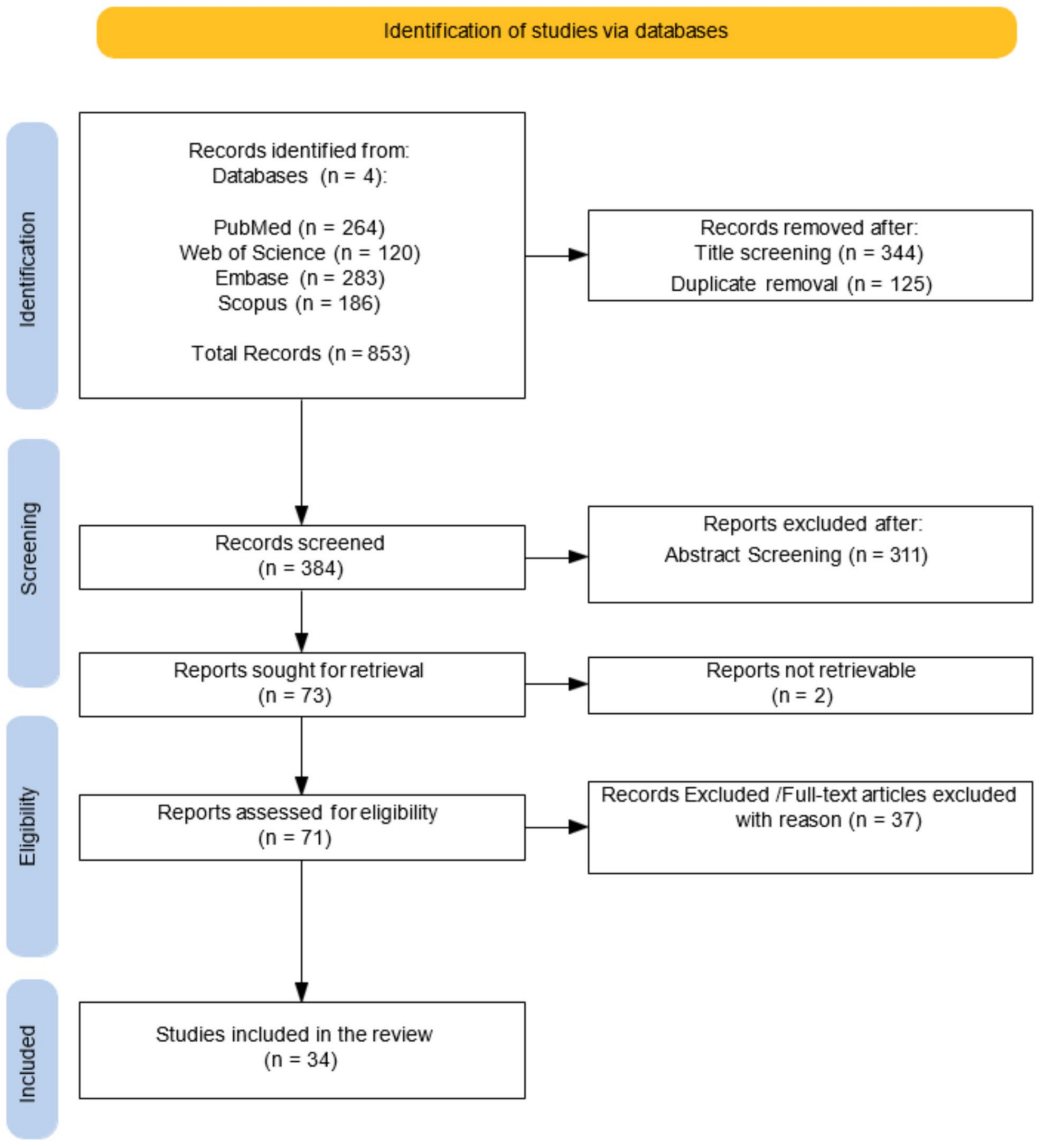
PRISMA flow diagram for research strategy.

A comprehensive search of databases yielded 853 initial studies: 264 articles from PubMed, 283 from Embase, 120 from Web of Science, 186 articles from Scopus. After screening the title database and removing duplicate studies, 384 publications were initially identified. On these, 71 studies were selected based on RRMS subjects. Following an accurate revision of full manuscripts, 34 articles satisfied the inclusion/exclusion criteria (Figure 1).

### 2.4 PECO evaluation

We employed a PECO (Population, Exposure, Comparison, Outcome) framework to structure our research question. The population consisted of adult patients (≥18 years) diagnosed with RRMS. The exposure was defined as WM microstructural alterations assessed through DTI parameters, including FA, MD, AD, RD and ADC. The comparison group included age- and sex-matched healthy controls with no history of neurological or psychiatric disorders. The outcome was cognitive performance, evaluated using a standardized battery of neuropsychological tests. This approach enabled us to explore the relationship between WM damage and cognitive functioning in RRMS subjects.

### 2.5 Assess the quality of included studies - risk of bias

**Figure 2:**
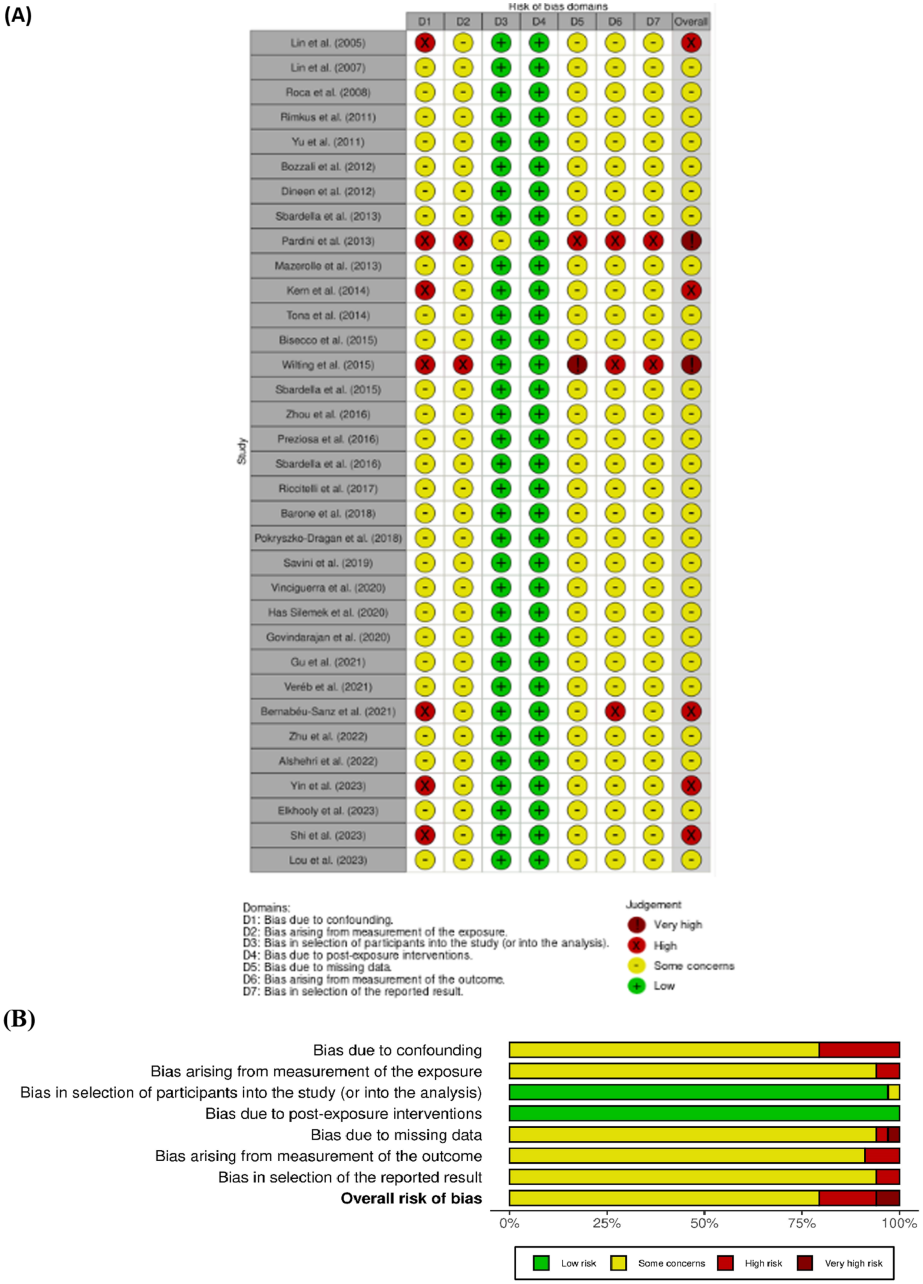
**(A, B)** ROBINS-E, summary of the risk of bias assessment and depicts distribution of bias concerns across the included studies.

The risk of bias in controlled studies was independently assessed by A. M. and C. S. any disagreements during this process, as well as during previous stages, were resolved through consultation with V. L. B., who provided the final decision. For all the selected study we used the Cochrane tool for non-randomized controlled studies-of exposures (ROBINS-E) tool (Higgins et al., 2024) (Figure 2), which comprises seven domains: (i) bias due to confounding, (ii) bias arising from measurement of the exposure, (iii) bias in selection of participants into the study (or into the analysis), (iv) bias due to post-exposure interventions, (v) bias due to missing data, (vi) bias arising from measurement of the outcome, (vii) bias in selection of reported result.

## 3 Result

### 3.1 Study characteristics

The main characteristics of all selected studies are summarized in Table 1. Of the 34 studies included, 20 were conducted in Europe (Pokryszko-Dragan et al., 2018; Bisecco et al., 2015; Bernabéu-Sanz et al., 2021; Has Silemek et al., 2020; Vinciguerra et al., 2021; Veréb et al., 2022; Sbardella et al., 2013, 2015, 2016; Savini et al., 2019; Tona et al., 2014; Riccitelli et al., 2019; Wilting et al., 2016; Bozzali et al., 2013; Pardini et al., 2014; Preziosa et al., 2016; Dineen et al., 2012; Barone et al., 2018; Lin et al., 2005, 2008), 7 in America (Rimkus et al., 2011; Mazerolle et al., 2013; Yu et al., 2012; Kern et al., 2014; Roca et al., 2008; Elkhooly et al., 2023; Govindarajan et al., 2021), 6 in China (Shi et al., 2023; Gu et al., 2022; Zhu et al., 2022; Zhou et al., 2016; Yin et al., 2023; Luo et al., 2024), and 1 in Australia (Alshehri et al., 2022).

**Table 1:** Summary of the included studies.

Author	Country	Aim of study	Study Design	Sample size (F:M); Age mean (SD)	EDSS (range)	MRI Device MRI strength	DTI Measure	ROI	Tests of neuropsicological evalutation	Key finding
Pokryszko-Dragan et al. (2018)	Poland	To assess damage to healthy WM and GM in specific brain regions and examine its association with specific aspects of cognition	Observational, cross-sectional	50 (37:13); Age 36.4	2.7(1–6)	1.5T Signa Hdx, GE Medical Systems	FA, ADC	Corpus Callosum, thalamus, cerebellum	PASAT SDMT (MSFC)	Significant microstructural alterations in the CC, thalamus, and middle cerebellar peduncle. FA values in the genu and splenium of the corpus callosum correlated positively with SDMT scores (*r* = 0.33, *p* = 0.022; *r* = 0.38, *p* = 0.009), while splenial ADC showed a negative correlation (*r* = –0.29, *p* = 0.046). Thalamic ADC on the right side was negatively associated with SDMT (*r* = –0.30, *p* = 0.041) and positively with 9HPT (*r* = 0.38, *p* = 0.008); left thalamic FA correlated negatively with 9HPT (*r* = –0.32, *p* = 0.031). ADC in the left middle cerebellar peduncle showed a positive correlation with 9HPT performance (*r* = 0.31, *p* = 0.032).
Alshehri et al. (2022)	Australia	To examine the relationships between DTI metrics and TBWM and WML in conjunction with clinical parameters in relation to volumetric assessments.	Observational, cross-sectional, case control study	37 (29:8); Age 37 (1, 21)	1.9 ± 0.15	3T Magnetom PRISMA system (Siemens Healthineers, Erlangen, Germany)	FA, MD, RD, AD	TBWM, WML	tARCS SDMT	RRMS subjects showed significant microstructural alterations TBWM, with increased MD (+3.6%, *p* ≤ 0.01), RD (4.8%, *p* ≤ 0.01), and AD (+2.7%, *p* ≤ 0.01), alongside decreased FA (−4.3%, *p* ≤ 0.01). Volumetric analysis revealed a reduction in total brain GM, and WM volumes (−5%, p ≤ 0.01), and an increase in cerebrospinal fluid volume (+26%, *p* ≤ 0.01). DTI metrics in RRMS showed moderate correlations with cognitive performance: FA in TBWM correlated positively with tARCS (*r* = 0.41), memory (*r* = 0.41), fluency (*r* = 0.35), and attention (*r* = 0.41), all *p* ≤ 0.05. RD in TBWM correlated negatively with tARCS (*r* = –0.43), memory (*r* = –0.47), and fluency (r = –0.45), all p ≤ 0.01. MD in TBWM showed negative correlations with memory (*r* = –0.37) and fluency (*r* = –0.39), both *p* ≤ 0.05; alterations in TBWM microstructure, with increased MD, RD, and AD values, and decreased FA.
Bisecco et al. (2015)	Netherlands, Austria, United Kingdom, Italy	To clarify the connection between damage to the thalamus and neuropsychological engagement and to examine the relationship between irregularities of regional thalamic MRI metrics and connectivity compared to mental capability.	Observational, cross-sectional, multi-center study with a case-control design	52 (33:19); Age 40, 3 (8, 5)	2 (0–6)	3Tat all sites (Amsterdam and Naples: GE) Signa; Graz and London: Siemens Trio; Milan and Siena: Philips Intera	DWI, FA, MD	Thalamus, cortico- thalamic tracts, cerebral volume	BRB-N SRT 10/36 SRT SDMT PASAT WLG WCST	RRMS subjects with CI showed increased FA in the frontal, motor, postcentral, and occipital regions connected to CDRs. They also had more pronounced atrophy in the anterior thalamic regions and abnormal DT MRI indices across all cortico-thalamic tracts including increased mean diffusivity (MD) in the frontal (*p* = 0.009), motor (*p* = 0.01), postcentral (*p* = 0.002), temporal (*p* = 0.0003), and occipital (*p* = 0.009) tracts, as well as decreased FA in the temporal (*p* = 0.007) and occipital (*p* = 0.008) tracts.
Bernabéu-Sanz et al. (2021)	Spain	This research explores the role of grey and WM in cognitive impairment in slightly disabled RRMS subjects.	Observational, cross-sectional, and case-control.	21 (9:21) Age 41	2 (0–2.5)	3T Philips Medical, The Netherlands	FA, MD	Brain Volumetry, VBM, TBSS	SDMT MAT global score MAT-orientation k z-MAT-semantic k z-MAT-free recall k z-MAT-cued-recall k z-MAT-episodic	In RRMS correlation between thalamic volume and SDMT scores was (*r* = 0.50, *p* = 0.005), and between FA in the left thalamus and SDMT (*r* = 0.40, *p* = 0.03). MD in thalamocortical projectionscorrelated with SDMT performance to the frontal (L: *r* = 0.55, *p* = 0.002; R: *r* = 0.56, *p* = 0.002), parietal (L: *r* = 0.58, *p* < 0.001; R: *r* = 0.55, *p* = 0.001), temporal (L: *r* = 0.55, *p* = 0.002; R: *r* = 0.57, *p* = 0.001), and occipital (L: *r* = 0.52, *p* = 0.003) cortices. WM damage at specific fasciculi was related to episodic memory impairment, with significant correlations observed between M@T episodic memory scores and streamlines in the left uncinate fasciculus (*r* = 0.57, *p* = 0.001), left inferior longitudinal fasciculus (*r* = 0.477, *p* = 0.009), and right cingulum (*r* = 0.38, *p* = 0.04).
Has Silemek et al. (2020)	Germany	To investigate the relationship between neuropsychological performance and both structural and functional networks in mildly disabled RRMS subjects in comparison to HC.	Observational, cross-sectional, and case-control.	33 (20:13); Age 40.9 (9, 7)	2 (0–4)	3T Skyra, Siemens Medical Systems, Erlangen, Germany	FA/FOD, MD	Structural and functional connectivity for each one of seven subnetworks based on the Yeo atlas	TAP PASAT SDMT VLMT WMS BVMT RWT	RRMS sshowed reduced global and WM brain volume compared to HC (*p* = 0.031 and *p* = 0.014, respectively). They also performed worse in short-term memory, verbal and visual learning, and executive functions. SDMT correlated positively with global structural graph strength (r = 0.46, *p* = 0.007), as did visuospatial memory scores from the BVMT Sum 1–3 (*r* = 0.55, *p* = 0.001) and BVMT Recall (*r* = 0.53, *p* = 0.002).
Vinciguerra et al. (2021)	Italy	To explore, the link between PSMD and cognitive performance, when compared to others MRI examination to explore if it can clarify cognitive performances more effectively than other measurements.	Observational, cross-sectional, and case-control.	60 (46:14); Age 43, 1 (9, 9)	1.5 (1–3)	3T	FA, MD	Brain volumetry, TBSS, Lesion volume	SDMT BRB-N SRT-LTS SRT-CLTR SRT-D SPART PASAT3 SPART-D WLG	PSMD showed a strong negative correlation with SDMT (*r* = −0.70, *p* < 0.001) and, to a lesser degree, with verbal memory tests such as SRT-LTS (*r* = −0.35, *p* = 0.02) and SRT-CLTR (*r* = −0.37, *p* = 0.016), as well as with visual memory (SPART): *r* = −0.28, *p* = 0.02 and verbal fluency (WLG): *r* = 0.25, *p* = 0.04. Multiple regression analysis indicated that PSMD accounted for 54% of the variance in SDMT (*R*^2^ = 0.54, *p* < 0.001), outperforming other MRI metrics, including WM skeleton MD (*R*^2^ = 0.30, *p* = 0.012).
Rimkus et al. (2011)	Brazil	To assess through DTI if the microstructure of the CC is already modified in MS subjects with a brief disease duration and to identify whether these DTI parameters correlate with the noted cognitive impairment.	Observational, cross-sectional, and case-control.	23 (15:18); Age 31, 95 (9, 2)	1.37 (0–4.5)	3T Intera Achieva, PHILIPS Healthcare, Best, The Netherlands	FA, MD, AD, VOI	Corpus Callosum	BVMT WAIS III SDMT TMT STROOP FAS WCST categori association HVLT im. Rec. HVLT de.rec. ROCFde.re.	RRMS subjects showed microstructural alterations in the CC, with reduced FA and increased MD, AD and RD, all significantly different from HC (MD: *p* < 0.0001; FA: *p* = 0.002; RD: *p* < 0.0001; AD: *p* = 0.009). AD was slightly higher in MS CI compared to MS CP (*p* = 0.048). CI showed significant correlations between FA and SDMT (*r* = 0.46, p = 0.02), RD and HVLT-delayed recall (*r* = 0.45, p = 0.03), and FA with Logical Memory II (*r* = 0.41, *p* = 0.05). Additional tendencies of correlation emerged between RD and SDMT (*r* = -0.37, *p* = 0.07), AD and TMT (*r* = -0.37, *p* = 0.08), and FA/RD with Stroop performance (*r* = 0.36, *p* = 0.08; *r* = −0.39, *p* = 0.06).
Luo et al. (2024)	China	To assess changes in diffusion kurtosis and susceptibility within the U-fiber area of individuals with RRMS and their links with cognitive status and degeneration.	Retrospective, cross- sectionl	49 (32:17) Age 32,88 (9, 64)	2.5 (2)	3T Magnetom Skyra, Siemens Healthcare GmbH	DKI - MK, AK, RK, KFA	Brain Volumetry, U- fiber.	MOCA DST SDMT	I In RRMS U-fiber lesion volume was positively correlated with fatigue (FSS: *r* = 0.329, *p* = 0.024) and depression (HAMD: *r* = 0.377, *p* = 0.008), and negatively correlated with cognitive performance (SDMT): (*r* = −0.335, *p* = 0.024). KFA values in both lesioned and non-lesioned U-fiber regions showed significant positive correlations with DST and SDMT scores (*r* = 0.371 and–0.399, *p* < 0.02), while increased magnetic susceptibility (mrQSM) in UFLs was negatively correlated with MoCA scores (*r* = −0.372, *p* = 0.021).
Veréb et al. (2022)	Hungary	To examine how FC within RSNs is asymmetrically distributed in patients with MS in comparison to a HC. Next, to assess if straying from the proper lateralization pattern reflects the cognitive performance.	Observational, cross-sectional, and case-control.	24 (18:6) Age 41, 18 (8, 85)	1 (0–3)	3 T GE MR750W Discovery scanner (GE, Milwaukee, USA)	FA	TBSS	BICAMS SDMT BVMT-R CVLT-II	RRMS showed significant leftward shift in functional lateralisation within the angular gyrus of the DMN (*p* < 0.005). This shift was negatively correlated with visuospatial memory performance, as assessed by BVMT-R, (*r*= –0.52, *p* < 0.023). DAN exhibited a significant reduction in rightward lateralisation in RRMS patients, particularly in the posterior intraparietal sulcus (*p* < 0.033). In contrast, HC demonstrated significant rightward dominance in this region (*p* < 0.001). The reduced lateralisation in the DAN was significantly associated with increased rightward asymmetry in FA within the superior longitudinal fasciculus (*p* < 0.02).
Sbardella et al. (2013)	Italy	To investigate the relationship between clinical disability and both GM and WM regional damage in MS subjects.	Observational, cross-sectional, and case-control.	36 (26:10) Age 34 (8)	2.5 (1–4.5)	3T Verio, Siemens AG, Erlangen, Germany	FA, MD, AD, RD	Resting State Networks, WBVA, TBSS	MSFC PASAT	in RRMS 9HPTND showed significant correlations with T2LV (*r* = 0.55, *p* = 0.001), FA (*r* = −0.41, *p* = 0.029), MD (*r* = 0.49, *p* = 0.003), AD (*r* = 0.49, *p* = 0.003), RD (*r* = 0.48, *p* = 0.004), and WM/ICV (*r* = −0.43, *p* = 0.012). PASAT 2s, was positively correlated with GM volume (GM/ICV ratio, *r* = 0.47, *p* = 0.005). VBM analysis revealed a significant correlation between GM volume and 9HPT in the cerebellum (lobules VIII–IX and left crus I, *p* < 0.05, FWE corrected), and between GM volume and PASAT in the orbitofrontal cortex bilaterally (*p* < 0.05, FWE corrected). TBSS revealed significant correlations between DTI metrics with 9HPT and PASAT scores in many WM bundles (*p* < 0.05, TFCE corrected), including the CC, internal capsule, posterior thalamic radiations, and cerebral peduncles.
Sbardella et al. (2015)	Italy	Finding RSNs abnormalities in MS and at investigating their correlations with both microstructural damage and clinical impairments. using RS-fMRI and DTI techniques in a cohort of RRMS subjects	Retrospective, Cross-sectional, case control	30 (21: 9) Age 35 (8.0)	2.5 (0–4)	3T Siemens Magnetom Verio (Siemens AG, Muenchen, DE).	FA, MD, AD, RD	WBVA, TBSS	PASAT MMSE MSFC	In individuals with RRMS, reduced FC was observed in 5 out of 11 resting-state networks, including the cerebellar, executive control, mid-visual, basal ganglia, and sensorimotor networks (*p* < 0.0001). Alterations were also found in inter-network correlations, alongside widespread microstructural damage in the white matter (*p* < 0.004). Microstructural damage in the CC was positively correlated with FC in the cerebellar and auditory networks (*p* < 0.004), suggesting a link between structural and FC. Meanwhile, FC within the mid-visual network was inversely associated with PASAT (*p* < 0.05).
Sbardella et al. (2016)	Italy	To evaluate changes in dentate FC among adults with RRMS and to explore potential clinical correlations.	Prospective, observational	54 (35: 19) Age 38, 55 (9, 46)	2.5 (1-5)	3T Siemens Magnetom Verio	FA	Dentate functional connectivity	PASAT (Z score)	FC in RRMS subjects correlated inversely with clinical impairment (*r* = −0.52, *p* < 0.001), particularly in the superior and middle frontal gyri, pre- and post-central gyri, and supplementary motor area (SMA) bilaterally. FC also correlated inversely with lesion load (*p* < 0.05, FWE corrected), with significant clusters in the cerebellum, right thalamus, and fronto-parieto-occipital cortices. FC correlated with PASAT (*r* = 0.43, *p* = 0.001), especially in the superior and middle frontal gyri, cingulate gyrus, and SMA on the right.
Mazerolle et al. (2013)	Canada	To examine the neural correlates of increased intra-individual variability (IIV) in RRMS individuals, focusing on its association with white matter microstructure. The study assessed mean reaction time and IIV on timed tasks, and investigated their relationship with SDMT performance, lesion burden, and global brain atrophy.	Retrospective, Cross-sectional, case control	20 female Age 42, 4 (6, 3)	2.25	1.5 T General Electric MRI	FA, MD, AD, RD	TBSS	BDI-Fast Screen SDMT	, RRMS showed significantly longer mean reaction times on the SRT (t (37.8) = 2.8; *p* < 0.01) and SSRT (t (38) = 3.2; *p* < 0.005) subtests, and significantly greater intra-individual variability (ISD) on the same subtests (SRT): t (38) = 3.03; *p* < 0.005; SSRT: (t (38) = 2.8; *p* < 0.01). Significant relationships between WM microstructure and IIV were observed in MS. Increased IIV on the SRT subtest was associated with reduced integrity in multiple WM tracts, including the corpus callosum (body, genu, splenium), posterior thalamic radiation, corona radiata, uncinate fasciculus, external capsule, and superior fronto-occipital fasciculus. Compared to slower information processing speed, as measured by mean CTIP response time or other neuropsychological test scores (e.g., SDMT), IIV demonstrated stronger and more widespread correlations with WM integrity. ISD on the SRT correlated significantly with lesion volume (*r* = 0.633; *p* = 0.004) and BPF (*r* = −0.484; *p* = 0.036), whereas SDMT performance correlated with fewer tracts and a weaker association with lesion volume (*r* = −0.533; *p* = 0.019).
Yin et al. (2023)	China	To utilize DTI and QSM to quantitatively assess the extent of microstructural damage and iron accumulation in both a cohort of MS patients and HCs; to integrate FA, MD, and QSV into a machine learning model using support vector machines to identify the most severely affected areas in MS patients and to examine the relationship between quantitative MRI metrics of the DGM in MS patients and clinical neurological scales.	Observational, cross-sectional, and case-control.	115 (72:12) Age 14, 43 (40, 57)	1.29	3T MR scanner (Magnetom Skyra, Siemens Healthcare GmbH, Erlangen, Germany)	FA, MD	Deep gray matter, Caudatete nucleus, putamen, globus pallidus, thalamus	MMSE SDMT MOCA	In RRMS the volume of the globus pallidus (GP) and thalamus showed positive correlations with SDMT scores (*r* = 0.289, *p* = 0.049; *r* = 0.365, *p* = 0.012, respectively), while the MD of the thalamuswas negatively correlated with SDMT (*r* = -0.419, *p* = 0.003). MoCA scores were positively correlated with the volume of the putamen, GP, and thalamus (*r* = 0.33, *p* = 0.024; *r* = 0.343, *p* = 0.018; *r* = 0.324, *p* = 0.026, respectively). EDSS were negatively correlated with the MD of the putamen and GP (*r* = -0.331, *p* = 0.023; *r* = -0.478, *p* = 0.001) and disease duration was negatively correlated with the volume of GP and thalamus (*r* = -0.477, *p* = 0.001; *r* = -0.406, *p* = 0.005) and positively with the MD of the thalamus (*r* = 0.427, *p* = 0.003). MVPA analysis demonstrated that DTI and QSM parameters could effectively discriminate MS patients from HC, with the CN emerging as the most informative region across all models. The MD-based classification model achieved the highest performance ((AUC) = 0.93, *p* = 0.001), followed by FA ((AUC) = 0.83, *p* = 0.001) and QSV ((AUC) = 0.81, *p* = 0.002).
Tona et al. (2014)	Italy	To assess functional alterations within the thalamic resting-state network may impact cognitive deficits in RRMS individuals.	Observational, cross-sectional, and case-control	48 (16:9) Age 36, 7 (8, 1)	2 (1–4.5)	3 T (Verio; Siemens, Erlangen, Germany)	FA, MD, AD, RD	Thalamocortical functional connectivity	PASAT	RRMS subjects showed GM and WM atrophy, in the cerebellum; basal ganglia; hippocampus; cingulum; and temporo-occipital, insular, frontal, and parietal cortices. They also exhibited significantly lower synchronization in the thalamus; cerebellum; cingulum; and insular, prefrontal, and parieto-occipital cortices (cluster level, *p* < 0.05, corrected for familywise error [FWE]). the PASAT 3s score significantly inversely correlated with the thalamus, cerebellum, and some cortical areas in all cerebral lobes (*p* < 0.05, FWE-corrected); the PASAT score at 2s seconds significantly correlated, with all the aforementioned regions and with the cingulum and the left hippocampus (*p* < 0.05, FWE-corrected).
Zhou et al. (2016)	China	To examine the functional and structural integrity of the thalamocortical system in subjects with RRMS.	Retrospective, Cross-sectional, case control	20 (15:5) Age 39, 35 (20–57)	1, 67 (0–2.5)	3T (Trio Tim, Siemens Medical Systems, Erlangen, Germany)	FA, MD, AD, RD	Thalamocortical system	PASAT	Thalamocortical connections of RRMS subjects showed lesion load-related regional FC in the right temporal pole. Significant correlations were observed between increased diffusivity and slowed cognitive processing as PASAT or the impact of fatigue (MFIS-5), as well as between connective fiber loss and disease duration.
Riccitelli et al. (2019)	Italy	To map the regional patterns WM microstructural abnormalities and GM atrophy that are specifically linked to decreased performance on the SDMT and the PASAT in patients with RRMS.	Retrospective, Cross-sectional, case control	177 (111: 66) Age 38, 3 (10, 5)	1,5 (1.5–3.0)	3T	FA, MD, AD, RD	Brain Volumetry, WBWM	PASAT SDMT	Compared to HC, RRMS showed significant WM microstructural abnormalities and GM atrophy (*p* < 0.05, FWE corrected). Lower SDMT scores correlated with longer disease duration (*r* = -0.20, *p* = 0.01), higher EDSS (*r* = -0.23, *p* = 0.002), reduced BPF (*r* = 0.21, *p* = 0.005) and WMF (*r* = 0.24, *p* < 0.002), but not GMF (*r* = 0.13, *p* = 0.09). SDMT performance was associated with decreased FA and increased MD, AD, RD in widespread WM tracts, and with GM atrophy in right anterior cingulate cortex (BA24; *r* = 0.23), left postcentral gyrus (BA4; *r* = 0.23), and right middle temporal gyrus (BA22; *r* = 0.22) (*p* < 0.001). Lower PASAT scores correlated with reduced BPF (*r* = 0.26, *p* < 0.0001), GMF (*r* = 0.16, *p* = 0.03), and WMF (*r* = 0.29, *p* < 0.0001). WM damage involved supratentorial tracts (*r* = 0.1–0.44), and GM atrophy affected right thalamus (*r* = 0.27), caudate (*r* = 0.37), putamen (*r* = 0.29), left pallidum (*r* = 0.31), right ACC, superior frontal gyrus (BA32/BA9; *r* = 0.26), precentral gyrus (BA6; *r* = 0.29), left STG (BA22; *r* = 0.28), and right fusiform gyrus (BA19; *r* = 0.30) (*p* < 0.001). Atrophy of left caudate, putamen, and thalamus correlated with both SDMT and PASAT scores.
Savini et al. (2019)	United Kingdom	To investigate the role of DMN in cognitive impairments associated with RRMS, with a focus on its structural connectivity with the cerebellum. The study aimed to determine whether structural alterations explain variability in information processing speed.	Cross-sectional, case-control design	68 (44: 22) Age 46, 7 (11, 0) CIMS (13: 7) Age 50.3 (10.6) CPMS (30:16) Age 45.3 (11.0)	4, 5 (1–8.5)	3T Philips Achieva MRI scanner (Philips Healthcare, Best, Netherlands)	FA	8 ROIs corresponding to the nodes of the resting state network and divided between right and left hemispheres: Left and right medial frontal cortex; Left and right angular gyrus; Left and right precuneus/posterior cingulate cortex; Left and right middle temporal gyrus	SDMT	Strongly correlation of CIMS SDMT scores with the FA-weighted global efficiency (GE) of the network GE (CBL-DMN) (*r* = 0.87, *R*^2^ = 0.76, *p* < 0.001), GE (DMN) (*r* = 0.82, *R*^2^ = 0.67, *p* < 0.001), and GE (CBL) (*r* = 0.80, *R*^2^ = 0.64, *p* < 0.001). In CPMS the correlation between these measures were significantly lower and SDMT scores correlated most with BPF. In a multivariable regression model where SDMT was the independent variable, FA-weighted GE was the only significant explanatory variable in CIMS (*p* < 0.001), while in CPMS BPF and EDSS were independently significant.
Yu et al. (2012)	USA	To employ a DTI analysis technique known TBSS, which offers enhanced sensitivity for identifying disruptions in WM tract integrity in individuals with RRMS.	Cross-sectional, case-control design	37 (31:6), Age 40.9 (10.1)	2.25 (0–4.4)	3T Phillips AchievaMR Scanner (Philips) Medical Systems, Best, The Netherlands	FA, MD, AD, RD	WBVA, WBWM, NAWM	PASAT SDMT RAVLT	In the RRMS group, voxel wise correlations were found between FA reduction and cognitive impairment in cognitively-relevant tracts, predominantly in the posterior thalamic radiation, the sagittal stratum, and the CC; strongest correlations were with SDMT measures (*p* < 0.01), with contributions to these associations from both lesion and normal-appearing white matter. Results using TFCE showed more widespread white matter involvement compared to cluster-based thresholding.
Wilting et al. (2016)	Germany	To explore the morphological and microstructural correlates, along with the neuropsychological parameters, of cognitive fatigue in patients with early RRMS. They sought to identify the anatomical brain regions linked to cognitive fatigue by utilizing VBM and TBSS.	Cross-sectional, case-control design	79 MS0 41 (29:15) MSF 38 (30:8) Age MS0 30 (17–54) MSF 34,5 (20–58) MSF 2 (0–10)	MS0 0.50 (0–3.5) MSF 1.50 (0–5.5)	3-T MR scanner (Magnetom Tim Trio, Siemens Healthcare, Erlangen, Germany)	FA, MD	WBVA, WBWM, Thalamus, Basal Ganglia, Frontal cortex	SDMT TAP PASAT VLMT dg5 VLMT dgt7 TMT-A TMT - B RWT	Showed higher MD in MSF patients. Strongly correlated for FA and MD values in the thalamus to non-fatigued patients (MS0), suggesting microstructural alterations in this region. Moreover, FA and MD values in the thalamus were found to be strongly correlated with SDMT (FA *p* = 0.032, *r* = 0.245; MD *p* = 0.006, *r* = −0.308), TMT-B (FA *p* = 0.007, *r* = 0.308), and overall cognitive impairment (MD *p* = 0.034, *r* = 0.242).
Kern et al. (2014)	USA	To assess multiple sclerosis-related damage to the hippocampal–thalamic–prefrontal network and to investigate the contribution of each component to distinct cognitive domains.	Cross-sectional, case-control design	27 (23: 4) Age (37, 9, 8.2)	2.5 (1.1)	3T Siemens Trio	FA, AD, RD	Cingulus, Uncinate fasciculus, thalamo hippocampus	BDI PASAT SDMT BSRT WAIS 7/24 SRT	T2 lesion volumes were significantly associated with BSRT learning performance (*R* = –0.54, *p* = 0.004) (worse performance with higher lesion volume) but not with PASAT, SDMT, or 7/24 learning scores. FA was significantly reduced in RRMS subjects in the cingulum (FA: 0.450 ± 0.052 vs 0.484 ± 0.030; p = 0.008), UF (0.386 ± 0.012 vs 0.411 ± 0.012; *p* = 0.005), and fornix (0.263 ± 0.061 vs 0.318 ± 0.036; *p* = 0.0004),as well as total hippocampal (8304 ± 689 mm^3^ vs 8819 ± 1090 mm^3^; *p* = 0.01) and thalamic volume (18,872 ± 2049 mm^3^ vs 20,945 ± 1286 mm^3^; *p* = 0.0001) compared to HC.
Bozzali et al. (2013)	Italy	Use of named ACM to assess structural connectivity modifications in RRMS patients and ascertain their relationship with the patients’ PASAT scores.	Retrospective, cross-sectional, case-control design	25 (19: 6) Age 34,5 (8.6)	2.0 (0.0–4.5)	3 T (Siemens Magnetom Allegra, Siemens Medical Solutions, Erlangen, Germany)	ACM, FA	WBVA, WBWM, WBACM	MSFC BRB PASAT correct PASAT error	RRMS subjects showed significant GM loss bilaterally in the post-central gyrus, insular cortex, parahippocampal gyrus, and amygdalae. RRMS patients displayed widespread reductions in FA. Voxel-wise comparison of ACMs revealed reductions in connectivity within the thalamus and CN bilaterally in RRMS patients compared to controls (p-FWE-corrected < 0.05). In RRMS subjects, better performance (more correct responses) on the PASAT-3s was directly correlated with higher ACM values in the anterior portion of the CC, the right hippocampus, and the cerebellum (p-FWE-corrected < 0.05).
Roca et al. (2008)	Argentina	To examine specific executive deficits in early stage of RRMS. The focus was to assess whether there was a potential correlation between fronto-subcortical DTI results and the presence of cognitive impairment.	Prospective recruitment, cross- sectional observational	12(6:6) Age 31,0 (8, 49)	< 2	1.5T (GE Medical System, Milwaukee, WI, USA).	FA, ADC	NABM in frontal lobe	PASAT(correct) MSFC MMSE ACE WAT.BA Vocabulary Subscale (WAIS) Raven Colour Progressive Matrices Paragraph Memory (immediate)-WMS Paragraph Memory (long-term)-WMS Recognition Rey list Rey figure Boston F.A.S Semantic Fluency Token Test Digits Forward Digits Backward TMT A TMT B Letters and Numbers WCST categories	MS subjets showed a significant increase in average ADC values in the fronto-medial (FM), fronto-lateral (FL), and gyrus cinguli (GC) areas (*p* = 0.01–0.006). FA values were significantly lower in MS patients in the FM and FL regions (*p* = 0.01–0.006), but not in the OF and GC areas. Significant positive correlation was found between PASAT performance and FA measures in the FL region (*r* = 0.64, *p* = 0.03). Correlations were observed between ADC measures in the FL region and the number of tasks achieved in the MET (*r* = 0.72, *p* = 0.01). A correlation was also found between ADC measures in the FL region and deviations from correct time in the Hotel Task (*r* = 0.68, *p* = 0.02).
Elkhooly et al. (2023)	USA	To deepen the relationships between WM and cognitive functions	Cross sectional, observational	30 (22:8) Age 43,83 (10, 28)	1.5 (1.5–3.4)	3-TSiemens Verio System (Siemens Medical Systems, Erlangen, Germany)	FA, MD	Brain Volumetry, TBWM	PASAT3 SDMT	In RRMS a significant correlation was found between nGMV and SDMT. A trend towards significance was observed between nGMV and SDMT (*r* = 0.563, *p* = 0.001), while a trend towards significance was observed between nGMV and PASAT-3 (*r* = 0.369, *p* = 0.05).Three regions with significant correlation were found between mean SDMT values and mean FA values in white matter tracts the left corticothalamic tract (*r* = 0.441, *p* = 0.015, FDR-adjusted *p* = 0.050), the superior cerebellar peduncle (*r* = 0.512, *p* = 0.004, FDR-adjusted *p* = 0.049), and the right medial lemniscus (*r* = 0.454, *p* = 0.012, FDR-adjusted *p* = 0.049). Three significant correlations were found between mean SDMT values and mean MD values in WM tracts the left corticothalamic tract (*r* = 0.441, *p* = 0.015, FDR-adjusted *p* = 0.050), the superior cerebellar peduncle (*r* = 0.512, *p* = 0.004, FDR-adjusted *p* = 0.049), and the right medial lemniscus (*r* = 0.454, *p* = 0.012, FDR-adjusted *p* = 0.049).
Pardini et al. (2014)	Italy	To clarify the potential roles of structural abnormalities of the hippocampus, UF and CB in both subjective and objective memory performance.	Retrospective, cross- sectional observational	25 (14:11) Age 30, 03 (8, 70)	1.48 (0.52)	1.5 T (Signa Excite; GE Healthcare, Milwaukee, WI)	FA	Hippocampal volume, FA for the Uncinate Fasciculus and for the ventral division of the cingulum bundle	BRB-N SRT D 10/36 SRT 7/24 SPART	In RRMS left NHV was significantly correlated with verbal memory performance (*r* = 0.52, *p* = 0.008), and right NHV with spatial memory performance (*r* = 0.56, *p* = 0.004). Additionally, reduced FA in the left VCB was significantly associated with higher subjective retrospective memory deficits (*r* = -0.53, *p* = 0.006), while reduced FA in the left UF correlated with increased subjective prospective memory complaints (*r* = -0.57, *p* = 0.003).
Preziosa et al. (2016)	Netherlands, Spain, Austria, United Kingdom, Italy	To evaluate the contribution of focal lesions, apparently normal WM, and GM damage to cognitive impairment in multiple sclerosis patients, using voxel-based analyses of structural MRI data.	Retrospective, cross- sectional observational	61(40:21) Age 39, 7 (8, 5)	1.5 (0–6)	3 T at all sites (Amsterdam and Naples: GE Signa; Barcelona, Graz, and London: Siemens Trio; Milan and Siena: Philips Intera)	FA, MD	Brain Volumes, WBWM	BRB-N SRT 10/36SRT SDMT PASAT WLG WCST	RRMS subjects showed reduced overall brain volume and signs of WM damage with significantly lower NBV (*p* < 0.0001), GMV (*p* < 0.0001), and WMV (*p* < 0.0001), alongside reduced FA (*p* = 0.0001) and increased MD in both WM (*p* = 0.03) and GM (*p* = 0.02). Cognitive impairment was linked to brain atrophy, with global cognitive impairment best predicted by FA reduction in the left SLF (*r* = 0.71, *p* < 0.001), MD increase in the left posterior corona radiata (*r* = –0.66, *p* < 0.001), and GM atrophy in the left postcentral gyrus (*r* = 0.53, *p* < 0.001) and right hippocampus (*r* = 0.59, *p* < 0.001).Attention and processing speed correlated with thalamic atrophy (*r* = 0.63, *p* < 0.001) and FA reduction in the ILF (*r* = 0.66, *p* < 0.001); executive dysfunction was linked to IFG atrophy (*r* = 0.65, *p* < 0.001) and FA reduction in the right IFOF (*r* = 0.68, *p* < 0.001); visual memory impairment correlated with MD increase in the splenium of the CC (*r* = –0.53, *p* < 0.001) and left ILF (*r* = –0.56, *p* < 0.001).
Zhu et al. (2022)	China	To assess microstructural damage in different WM matter tissue types in RRMS and HC, and to examine the relationship with cognitive performance to explore their potential as imaging biomarkers of disease severity.	Retrospettive, cross-sectional, case-control design	48 (31:17) Age 33, 1 (9, 2)	2.2 (1.3)	3-T (Magnetom) Skyra, Siemens Healthcare GmbH, Erlangen, Germany	DKI-KFA, FA, MK, MD	Whole Brain Lesion Volume, NAWM, WBWM	MMSE MOCA SDMT	DKI parameter: T1Ls showed the lowest FA and MK values and the highest KFA and MD values, followed by pure-T2Ls, NAWM, and WM in controls. MK valuesof pure-T2Ls were correlated with MMSE scores. KFA, FA, MK, and MD values of NAWM were correlated with MMSE scores. Pure-T2L FA values correlated with MoCA scores.
Dineen et al. (2012)	United Kingdom	To determine the relative contribution of reduced volumes of the hippocampus, thalamus, and mammillary bodies, along with decreased integrity of the fornical tract, to performance on cognitive tests.	Prospective, cross-sectional, case-control design	34 (23:11) Age 42, 6 (31.1–56, 1)	2.5 (1.5–6.5)	3T Achieva, Philips, Eindhoven, NL	FA, AD, RD	Fornix regions, hippocampal, mammillary body and thalamic	Zscore of: BVRT CVLTII CVLT-II recognition COWAT	In RRMS a reduction in fornix FA (mean adjusted for age and volume): 0.37 vs 0.46; (*F* (1, 55) = 34.6, *p* < 0.0005), increased fornix RD, and significant reduction in mammillary body volume (0.114 ml vs 0.126 ml; F (1, 56) = 5.5, *p* = 0.023). Fornix FA showed a significant positive correlation with thalamic volume across all subjects (*R* = 0.45, *p* < 0.0005) and within the MS group (*R* = 0.55, *p* < 0.0005), but no correlation with hippocampal volume was found. Furthermore, fornix FA and mammillary body volume significantly predicted visual recall performance (*R*^2^ = 0.31, *p* = 0.008 and p = 0.038, respectively).
Govindarajan et al. (2021)	USA	To investigate WM correlates of slowed information processing speed in young subjects with developmental-onset RRMS, correlating.	Cross-sectional, case-control design.	25 (14:11) Age 20,8 (4, 5) Age 15 (3, 7)	5.7 (3.6)	3 T Siemens Biograph mMR	FA, MD, AD, RD	Focal WM Lesions, WBWM, Specific White Matter Tracts (Acoustic radiation Callosal body, Cingulum Corticospinal tract, Fornix, Inferior occipito-frontal fascicle, Optic radiation, Superior longitudinal fascicle, Superior occipito-frontal fascicle, Uncinate fascicle).	Z score of SDMT Cogstate brief battery: Detection (DET) Identification (IDN)	RRMS showed significant slowing of reaction times in some tasks of the CBB, the DET and the IDN. DTI analysis revealed widespread and marked WM abnormalities manifested by lower FA and higher MD, RD and AD compared to HC. Increased MD, RD, and AD in the corpus callosum and left corticospinal tract were significantly and negatively correlated with SDMT scores (*p* < 0.05, corrected), while MD in the right superior occipitofrontal fascicle showed a significant negative correlation with DET scores (*p* < 0.05, corrected).
Barone et al. (2018)	Italy	To investigate whether the integrity of normal-appearing CC WM influences clinical and neuropsychological features in RRMS	Retrospective observational, cross- sectional	74 (44: 30) Age 34,8 (9,5)	2 (2-3,8)	3-T GE MR750 scanner (GE Healthcare, Rahway, NJ, USA).	FA	Corpus Callosum	MMSE BRB-N	Three distinct RRMS clusters were identified based on CC integrity: Cluster 1: RRMS with CC integrity comparable to HC; showed near-normal cognitive performance, with only mild deficits in verbal fluency (*p* < 0.001 vs HC). Cluster 2: RRMS with mild CC damage; exhibited moderate cognitive impairments, more pronounced than Cluster 1 but less severe than Cluster 3. Significant deficits were observed in executive functions (SDMT), (*p* = 0.05 vs Cluster 1; *p* = 0.004 vs HC) and processing speed (STROOP-C), (*p* = 0.05 vs Cluster 1; *p* = 0.04 vs HC). Verbal fluency was also significantly reduced (*p* < 0.001 vs HC). Cluster 3: RRMS with the most severe CC damage; had the longest disease duration, highest EDSS scores, and greatest lesion load (global and callosal). This group showed widespread cognitive deficits, including impairments in verbal memory (SRTD), (*p* = 0.05 vs Cluster 1; *p* = 0.02 vs HC), verbal fluency (*p* < 0.001 vs HC), executive functions (SDMT), (*p* = 0.007 vs Cluster 1; *p* < 0.001 vs HC), and processing speed (STROOP-C), (*p* = 0.05 vs Cluster 1; *p* = 0.04 vs HC). Overall cognitive performance (cognitive score, *p* = 0.005 vs Cluster 1; *p* < 0.0001 vs HC).
Lin et al. (2005)	United Kingdom	The study aimed to identify pyramidal tracts and CC in patients with MS and assess whether this correlated with disability and cognitive performance scores.	Retrospective cross- sectional	29 (22: 7) Age 38 (31 – 41)	2.5 (2–3.25)	1.5-T Magnetom Vision, Siemens, Erlangen, Germany	ADC	Pyramidal tracts, Corpus Callosum	PASAT	ADCav of pyramidal tracts and CC was significantly higher in RRMS compared to HC. A correlation was found between the ADCav of pyramidal tracts and pyramidal function system score. The ADCav of CC showed a significant correlation with PASAT. The overall T2 lesion volume did not correlate with EDSS. However, it significantly correlated with the ADC in pyramidal tracts and CC. The overall T2 lesion volume also correlated with PASAT. The T2 lesion volume in pyramidal tracts correlated with the ADCav of pyramidal tracts and with the overall T2 lesion volume. Finally, the T2 lesion volume in CC significantly correlated with the ADC of CC and with the overall T2 lesion volume.
Lin et al. (2008)	United Kingdom	To explore the relationship between CC integrity and processing speed in RRMS.	Retrospective, Observational/cross sectional	36 (26:10) Age 37,5 (7,15)	3 (1.3)	1.5 T imaging system (Magnetom Vision, Siemens, Erlangen, Germany)	ADC	Corpus callosum	PASAT	The CC area was significantly reduced in cognitively impaired RRMS subjects compared to both HC (*p* = 0.02) PASAT scores were significantly correlated with CC magnetisation transfer ratio (*r* = 0.47, *p* = 0.0046), mean apparent diffusion coefficient (*r* = -0.53, *p* = 0.0012), CC area (*r* = 0.42, *p* = 0.01), and total T2 lesion load (*r* = -0.40, *p* = 0.017)
Gu et al. (2022)	China	To investigate the relationship between structural and functional alterations in the hippocampus and cognitive deficits.	Retrospective, cross-sectional, case-control design.	20 (15:5) Age 43 (12, 5)	2.50 (1–4.5)	3T Siemens Medical Systems, Erlangen, Germany	FA, MD	WBWM, Hippocampus	MOCA	In RRMS subjects whole-brain FC in the left hippocampus showed a negative correlation with the MoCA score. Whole-brain FC in the right hippocampus showed a negative correlation with the EDSS score. The MD value in the left hippocampus was negatively correlated with the MoCA score and positively correlated with the EDSS score.
Shi et al. (2023)	China	To explore whether diffusion parameters (KFA, FA, MK, and MD) can differentiate the WM tissue, evaluate the microstructure damage between different types of MS lesions and their PLWM and the results of diffusion parameters with the cognitive state and clinical biomarkers of disability.	Retrospective, cross-sectional, case-control study	68 (56: 12) Age 29.9 (9.3)	1.4 (1.5)	3T MAGNETOM Skyra, Siemens, Erlangen, Germany	KFA, FA, MK, MD	CELs, non-CELs, NAWM, Perilesional WM	SDMT MMSE MOCA	Lower FA values in the bilateral hippocampus of RRMS subjects, while the MD values in the left hippocampus were significantly higher (*p* < 0.05). The MD value of the left hippocampus showed a significant negative correlation with the MoCA score. KFA of the perilesional WM of enhancing lesions correlated with the SDMT score. Both the KFA (*r* = 0.443, *p* = 0.021) and MD (*r* = –0.518, *p* = 0.006) of the perilesional WMof iron rim lesions (IRLs-PLWM) were significantly correlated with the DST score.

The studies collectively included data from 1,460 individuals with RRMS, of whom 72% were female. The average age was 35.7 ± 6.68 years, and the mean disease duration was 9.99 ± 6.68 years.

#### 3.1.1 Correlation between diffusion tensor imaging metrics and cognitive function

This section describes the relationship between alteration in DTI parameters and cognitive performance according to specific anatomical structures, including global WM and network connectivity, CC and commissure, thalamus, hippocampus, cerebellum, cingulum, and cerebral fascicles. This structure-oriented analysis provides a more detailed understanding of microstructural WM changes in cognitive outcomes in individuals with RRMS.

#### 3.1.2 Global WM and network connectivity

Nine studies showed a significant correlation between DTI parameters and cognitive performance, emphasizing a consistent association between WM integrity and cognition in RRMS. Alshehri et al. (2022) found that total Fractional Anisotropy Total Brain White Matter (FA-TBWM) showed moderate positive correlations with total Audio Recorded Cognitive Screen (tARCS) (*r* = 0.41, *p* ≤ 0.05), memory (*r* = 0.41, *p* ≤ 0.05), fluency (*r* = 0.35, *p* ≤ 0.05), and attention (*r* = 0.41, *p* ≤ 0.05) and positive but non-significant correlation with SDMT scores. Conversely, Mean Diffusivity Total Brain White Matter (MD-TBWM) correlated negatively with memory (*r* = −0.37, *p* ≤ 0.05) and fluency (*r* = −0.39, *p* ≤ 0.05), while RD-TBWM showed stronger negative correlations with tARCS (*r* = −0.43, *p* ≤ 0.01), memory (*r* = −0.47, *p* ≤ 0.01), and fluency (*r* = −0.45, *p* ≤ 0.01). Enhanced structural connectivity within the default mode network (DMN) showed a positive correlation with improved performance in verbal memory (VLMT) and spatial memory (BVMT), as well as in attention tasks (PASAT and TAP). A higher global structural strength was associated with better performance in SDMT (*r* = 0.46, *p* = 0.007) and BVMT (Sum 1–3: *r* = 0.55, *p* = 0.001 and Recall: *r* = 0.53, *p* = 0.002) across most functional networks (Yeo networks), particularly in DMN. Similarly, VLMT scores were positively correlated with a higher global structural strength within the nodes of the DMN and the limbic network (*p* < 0.05) (Has Silemek et al., 2020). Global Efficiency (GE) of FA-weighted networks correlated strongly with SDMT, indicating that greater damage corresponded to worse cognitive performance. In RRMS subjects with cognitive dysfunction, GE within the DMN was strongly associated with SDMT (*r* = 0.87, *R*^2^ = 0.76, *p* < 0.001), while this correlation was significantly weaker in cognitively preserved RRMS subjects (Savini et al., 2019). Veréb et al. (2022) found that reduced rightward lateralization of DMN areas, particularly in the angular gyrus and inferior parietal lobule *p* < 0.005, was correlated with better scores on the Brief Visuospatial Memory Test-Revised (BVMT-R) (*r* = −0.52, *p* < 0.023). Vinciguerra et al. (2021) reported moderate correlations between peak skeletonized mean diffusivity (PSMD) and verbal memory (SRT-LTS: *r* = −0.35, *p* = 0.02, SRT-CLTR: *r* = −0.37, *p* = 0.016), visual memory (SPART): *r* = −0.28, *p* = 0.02 and verbal fluency (WLG): *r* = 0.25, *p* = 0.04. As well as strong associations between both MD and FA of the WM skeleton (*R*^2^ = 0.30, *p* = 0.012) and SDMT (*R*^2^ = 0.54, *p* < 0.001), confirming the relationship between WM microstructural integrity and processing speed. In individuals with RRMS, KFA values of both U-fibre lesions (UFLs) and non-UFLs showed positive correlations with Digit Span Test (DST) (*r* = 0.371, *p* < 0.02) and Symbol Digit Modalities Test (SDMT) scores (*r* = −0.399, *p* < 0.02). Additionally, increased magnetic susceptibility (mrQSM) in UFLs was negatively correlated with Montreal Cognitive Assessment (MoCA) scores (*r* = −0.372, *p* = 0.021), suggesting a link between lesion burden and global cognitive decline. U-fibre lesion volume also showed a negative correlation with SDMT performance (*r* = −0.335, *p* = 0.024) (Luo et al., 2024). Elevated MK in lesions correlated with reduced MoCA and SDMT scores, whereas FA in T1 and T2 lesions showed a relationship with MoCA and SDMT scores. MD values showed a negative correlation with cognitive scores, suggesting a link between WM alterations and cognitive function (Zhu et al., 2022). Finally, the KFA values of PLWM linked to Contrast Enhancement Lesions (CELs) showed a positive correlation with SDMT scores. Furthermore, in PLWM associated with iron rim lesions (IRLs), both KFA and MD values were significantly correlated with DST scores: KFA showed a positive correlation (*r* = 0.443, *p* = 0.021), while MD showed a negative correlation (*r* = −0.518, *p* = 0.006). These findings suggest that microstructural damage in IRL-associated regions is linked to reduced working memory performance in RRMS (Shi et al., 2023).

Overall, these findings highlight a consistent relationship between WM microstructural integrity and cognitive performance in RRMS, indicating that widespread and network-specific alterations, particularly within the DMN and limbic circuits, contribute to deficits in processing speed, memory, and executive functioning.

### 3.2 Corpus callosum and commissure

Seventeen studies reported significant associations between DTI metrics of the CC and cognitive performance in RRMS. FA measures in the genu and splenium of the CC showed positive correlation with SDMT performance (*r* = 0.33, *p* = 0.022 for the genu; *r* = 0.38, *p* = 0.009 for the splenium) and negative correlation with SDMT performance and negative correlation with Apparent Diffusion Coefficient (ADC) in the splenium (*r* = −0.29, *p* = 0.046) (Pokryszko-Dragan et al., 2018). Streamlined tractography analyses further revealed a positive association between SDMT scores and FA in occipital callosal fibres (*r* = 0.52, *p* = 0.003) (Bernabéu-Sanz et al., 2021). FA and RD were correlated with SDMT (FA: *r* = 0.46, *p* = 0.02; RD: *r* = −0.37, *p* = 0.07) and the Hopkins Verbal Learning Test–Delayed Recall (HVLT-DR) performance (*r* = 0.45, *p* = 0.03). Additionally, FA in the corpus callosum correlated with Logical Memory II (*r* = 0.41, *p* = 0.05), and RD with Stroop performance (*r* = −0.39, *p* = 0.06) (Rimkus et al., 2011). Similar FA, MD, AD, and RD measures have all shown a positive correlation with performance on the PASAT-2 s (GM/ICV ratio, *r* = 0.47, *p* = 0.005) (Sbardella et al., 2013, 2015). Lower FA and higher MD, RD, and AD values have been consistently associated with poorer SDMT performance and slower response times, including on tasks such as the Simple Reaction Time (SRT) and Semantic Search Reaction Time (SSRT) and Semantic Search Reaction Time (*p* < 0.05) (Mazerolle et al., 2013). Other research has shown that reduced SDMT and PASAT scores are linked to increased MD and AD values (PASAT: *r* = 0.26, *p* < 0.0001; SDMT: *r* = −0.20, *p* = 0.01) (Riccitelli et al., 2019), as well as elevated average ADCa values in relation to PASAT performance (*r* = −0.53, *p* = 0.0012) (Lin et al., 2005, 2008). Moreover, decreased FA in callosal fibers has been associated with impairments on Rey Auditory Verbal Learning Test (RAVLT), PASAT, and most notably, the SDMT (Yu et al., 2012). Positive correlations have also been observed between anterior commissure microstructural integrity and PASAT-3 s scores in CC areas (Bozzali et al., 2013). Govindarajan et al. (2021) did not find a significant correlation between FA and cognitive performance. However, MD, RD, AD were negatively correlated with SDMT scores (*p* < 0.05), in addition to correlations between MD, AD, and reaction time on the Detection task (DET). Barone et al. (2018) further stratified RRMS subjects into Cluster in based on FA and CC volume, showing that Cluster 2 (mild CC damage) had significant deficits in verbal fluency, executive function, processing speed measured with Word List Generation test (WLG), SDMT, STROOP-C (*p* = 0.05 vs. Cluster 1; *p* = 0.004 vs. HC), and Cluster 3 (patients with the most severe CC damage) had lowest scores in this valuation and also in SRTD for verbal memory (*p* = 0.05 vs. Cluster 1; *p* = 0.02 vs. HC). Similarly, heightened MD and reduced FA in the splenium of the CC were associated with deficits in visual memory, attention, and executive functions score of 10/36 Spatial Recall Test (10/36 SRT), SDMT, PASAT and Wisconsin Card Sorting Test (WCST) instead for splenium of the CC the decreased FA correlated with 10/36 Spatial Recall Test, SDMT and PASAT, and increased MD with WCST, SDMT and PASAT. Also, in Forceps Major and Minor decreased FA correlated with SDMT and PASAT, 10/36 SRT and MD increased with 10/36 SRT, SDMT and PASAT (Preziosa et al., 2016). Finally, Dineen et al. (2012) showed that a reduction of fornix FA had a significant correlation with low scores in BVRT (Benton Visual Retention Test) (*R*^2^ = 0.31, *p* = 0.008).

Overall, these findings suggest that microstructural disruption within the CC, reflected by decreased FA and increased diffusivity, likely represents impaired interhemispheric communication, contributing to reduced information processing speed and global cognitive efficiency. The observed correlations highlight the central role of callosal integrity in sustaining distributed cognitive networks in RRMS.

#### 3.2.1 Thalamus

A total of ten studies have reported correlations between the microstructural characteristics of thalamocortical WM pathways and cognitive alterations in RRMS subjects. ADC measurements have shown a strong correlation with SDMT scores (*r* = −0.30, *p* = 0.041) (Pokryszko-Dragan et al., 2018). Bisecco et al. (2015) found that attention, spatial and verbal memory, verbal fluency, and executive functions, measured with several tests BRB-N, SRT, 10/36, SDMT, PASAT 2 and 3, Word List Generation (WLG), Wisconsin Card Sorting Test (WCST), were predicted by lesion volume or MD within corticothalamic tracts. Verbal memory correlated with MD in bilateral fronto-caudate-radial (F-CDR) pathways, spatial memory related to MD in the left thalamo-temporal (T–T) pathway, and verbal fluency was linked to both FA in the occipital-caudate-radial (O-CDR) pathway and MD in the left fronto-thalamic (F-T) pathway. Executive functions were associated with MD in the right F-CDR pathway. Bernabéu-Sanz et al. (2021) found that SDMT scores were positively correlated with FA (*r* = 0.40, *p* = 0.03) and negatively with MD in bilateral thalamic projections to the frontal (left: *r* = 0.55, *p* = 0.002; right: *r* = 0.56, *p* = 0.002), parietal (left: *r* = 0.58, *p* < 0.001; right: *r* = 0.55, *p* = 0.001), temporal (left: *r* = 0.55, *p* = 0.002; right: *r* = 0.57, *p* = 0.001), and occipital cortices (left: *r* = 0.52, *p* = 0.003). Tona et al. (2014) found that increased thalamic functional connectivity was inversely related to PASAT performance (*p* < 0.05), whereas no significant correlations were found between FA, MD, or thalamic volumes and PASAT. Zhou et al. (2016) found a negative relationship between AD in the prefrontal corticothalamic tract and PASAT performance. In line with these findings, Wilting et al. (2016) showed that FA and MD values in the thalamus were significantly related to processing speed, cognitive flexibility measured by SDMT, and TMT (TMT-B: FA *r* = 0.308, *p* = 0.007; MD *r* = 0.242, *p* = 0.034). Additionally, MD values in the thalamus were significantly correlated with overall cognitive impairment. Moreover, Yin et al. (2023) validated a negative relationship between thalamic MD and SDMT performance (*r* = −0.419, *p* = 0.003). Mazerolle et al. (2013) offered additional evidence, associating diminished FA and elevated MD, RD, and AD in thalamic radiations with lower SDMT scores and extended reaction times in both simple and semantic tasks. In addition, Preziosa et al. (2016) showed that decreased FA of thalamic radiation and increased MD were correlated with visual memory (10/36 SRT *r* = −0.53, *p* < 0.001), attention, and processing speed such as SDMT and PASAT (*r* = 0.63, *p* < 0.001 for thalamic atrophy). Recently, Elkhooly et al. (2023) noted stable patterns between FA in distinct corticothalamic pathways and SDMT performance (*r* = 0.441, *p* = 0.015), highlighting the importance of thalamocortical connectivity in cognitive function in RRMS people. In summary, these findings suggest that microstructural alterations within thalamocortical pathways, as reflected by diffusion metrics such as FA, MD, and ADC, are strongly associated with cognitive decline in RRMS, particularly affecting processing speed, memory, and executive functions.

#### 3.2.2 Hippocampus

Regarding the role of hippocampal structures, Gu et al. (2022) found a significant negative correlation between MD in the left hippocampus and MoCA scores. Additionally, Tona et al. (2014) identified a significant inverse relationship between FC in the right hippocampus and PASAT-2 s performance (*p* < 0.05). These findings underscore the critical role of hippocampal integrity and commissural pathways in supporting cognitive function in MS.

#### 3.2.3 Cerebellar structure

Significant correlations have been observed between cognitive abilities and microstructural characteristics of cerebellar WM in 2 studies. Elkhooly et al. (2023) reported significant negative correlations between mean diffusivity (MD) values and SDMT scores, particularly in white matter regions such as the superior cerebellar peduncle (*r* = −0.512, *p* = 0.004), the left corticothalamic tract (*r* = −0.441, *p* = 0.015), and the right medial lemniscus (*r* = −0.454, *p* = 0.012). In contrast, fractional anisotropy (FA) values in these regions showed positive correlations with SDMT scores, suggesting that preserved microstructural integrity in cerebellar efferent pathways may support faster cognitive processing. Likewise, Riccitelli et al. (2019) noted that decreased FA in the middle and superior Cerebellar Peduncle (CP) and RD increased in left middle, inferior, and superior CP were significantly with poorer SDMT performance (*p* < 0.05) while Sbardella et al. (2013) showed positive associations between FA, MD, and PASAT-2 s scores across various WM tracts as the cerebral peduncles (*p* < 0.05). These studies emphasize the role of cerebellar and brainstem WM structures in cognitive efficiency while highlighting their susceptibility to cognitive impairments associated with RRMS subjects.

#### 3.2.4 Cingulum and cerebral fascicles

Five selected studies investigated the correlations between cognitive test performance and the microstructural integrity of the cingulate and cerebral WM fasciculi. SDMT scores, significant positive correlations with streamline integrity and negative with AD in the right inferior fronto-occipital fasciculus (IFO) (Bernabéu-Sanz et al., 2021). In the same study, Multiple Ability Test subtests were associated with streamlines in the left uncinate fasciculus (UF) *r* = 0.57, *p* = 0.001 and left inferior longitudinal fasciculus (ILF) (ILF; *r* = 0.477, *p* = 0.009), right cingulum (*r* = 0.38, *p* = 0.04), along with FA and AD in the fornix in relation to episodic memory performance. Kern et al. (2014) found associations between better WM integrity and higher scores in neuropsychological assessment. In particular, processing speed was positively associated with greater integrity of the UF integrity, with FA-RD relationship drove the association, and a similar pattern was observed for spatial memory. Sbardella et al. (2013) MD, AD, and RD showed positive correlations with PASAT 2 s scores in cingulum (*p* < 0.05) confirmed in subsequent studies (Sbardella et al., 2016). The comprehensive analysis by Preziosa et al. (2016) found that decreased FA of the left superior longitudinal fasciculus (SLF) (*r* = 0.71, *p* < 0.001) and right cingulate were predictive of global cognitive dysfunction defined as having abnormal performance on at least two tests of the BRB-N battery and WCST. In SLF, Inferior fronto-occipital fasciculus (IFOF) and right cingulate increased MD and decreased FA correlated with worse visual memory (10/36 SRT), attention and processing speed (SDMT and PASAT) and executive function (WCST). Moreover, in the UF increased MD correlated with worse scores in SDMT, PASAT, 10/36 SRT while decreased FA with worse WCST.

DTI parameters as MD, RD, and AD were negatively correlated with SDMT scores in the left corticospinal tract (*p* < 0.05). MD was also negatively correlated with DET scores in the right superior occipital-frontal fasciculus (*p* < 0.05). RD showed a significant negative correlation with DET scores in the left superior longitudinal fasciculus, with correlations observed for AD and RD in small clusters in the right superior occipital-frontal fasciculus (Govindarajan et al., 2021).

In summary, the evidence consistently indicates that microstructural disruptions within the cingulate and major cerebral WM fasciculi, particularly the SLF, UF, ILF, IFOF, and cingulum, are closely linked to cognitive impairment in RRMS. Alterations in diffusion metrics (FA, MD, AD, RD) across these tracts are systematically associated with deficits in processing speed, memory, attention, and executive functioning, emphasizing the critical contribution of fronto-limbic and associative pathways to cognitive integrity in this population.

#### 3.2.5 Other cerebral WM tracts

Five studies described the connection between WM and cognitive functioning in different neuropsychological measures. ADC values were positively correlated with performance on the Multiple Errands Test – Hospital Version (MET) (*r* = 0.72, *p* = 0.01) and the Hotel Task (r = 0.68, *p* = 0.02), particularly in the fronto-lateral (FL) region (Roca et al., 2008). Furthermore, a positive correlation between FA and PASAT 2 s scores was observed in the internal capsule. Similarly, Preziosa et al. (2016) reported that decreased FA in corona radiata correlated with poorer performance on SDMT and PASAT and 10/36 SRT, while increased MD in the same region was linked to 10/36 SRT.

In a previous study, Sbardella et al. (2013) showed positive associations between FA, MD, and PASAT-2 s scores across various WM tracts as the internal and external capsule (*p* < 0.05).

Direct associations were identified between the connectivity of the dentate gyrus with the frontal cortex and PASAT performance (Sbardella et al., 2016). Additionally, reductions in FA were significantly correlated with performance on the SDMT, RAVLT, and PASAT, with the strongest association found for the SDMT. In associative fibers such as the cingulum and the superior longitudinal fasciculus, FA reductions correlated with SDMT and PASAT. Similarly, in projection fibers such as the corona radiata and the internal capsule, significant correlations were found with SDMT, RAVLT, and PASAT (*p* < 0.01) (Yu et al., 2012).

These studies collectively demonstrate that disruptions in WM microstructure, particularly within projection (e.g., corona radiata, internal capsule, cerebral peduncles) and associative tracts (e.g., cingulum, superior longitudinal fasciculus), are strongly associated with cognitive impairment in RRMS. Alterations in diffusion metrics, including FA, MD, AD, and RD, consistently correlate with deficits in processing speed, memory, and executive functioning, highlighting the crucial role of widespread fronto-subcortical and cerebellar connections in supporting cognitive efficiency.

Across studies, alterations in WM microstructural integrity, particularly as measured by FA, MD, and RD, were consistently associated with cognitive performance in individuals with RRMS. Higher FA in key commissural tracts, such as the CC, and in subcortical structures, including the thalamus and hippocampus, generally correlated with better processing speed, attention, memory, and executive function. Conversely, increased MD and RD, especially in regions affected by U-fiber or other focal lesions, were associated with poorer cognitive outcomes.

A comprehensive overview of these correlations is provided in Supplementary Table 1 (overview of reported correlations), while structure-specific associations between DTI metrics and cognitive performance are detailed in Supplementary Table 2.

### 3.3 Risk of bias

The Cochrane Risk of Bias Assessment Tool (ROBINS-E) (Barone et al., 2018) was used to assess the risk of bias of the articles included in this review. Figure 2 shows the summary of the risk of bias assessment, while the graph depicts the distribution of bias concerns across the included studies. Most of the studies reviewed were observational and cross-sectional, and therefore, they were reported as having “some concerns” about bias (Lin et al., 2008; Roca et al., 2008; Yu et al., 2012; Bozzali et al., 2013; Sbardella et al., 2013, 2015, 2016). Common issues included inadequate adjustment for confounding variables, non-random selection of participants, use of retrospective designs, or data collection at a single point in time. Fewer studies were rated as having “high” risk of bias indicating more serious problems (Kern et al., 2014; Bernabéu-Sanz et al., 2021; Yin et al., 2023; Shi et al., 2023). These issues included a lack of clarity in measuring exposures and outcomes, poor communication of methods, and insufficient control for differences between participants that could influence the results. Two studies (Pardini et al., 2014; Wilting et al., 2016) were rated as having a “very high risk of bias,” reflecting significant methodological limitations such as heterogeneous outcome measures, inadequate sample representativeness, and incomplete reporting.

## 4 Discussion

The findings summarized in this review underscore the critical role of WM integrity, as measured by DTI, in sustaining cognitive function in individuals with RRMS. Across numerous studies, alterations in DTI metrics, particularly FA, but also MD, and RD, have consistently been associated with impairment in cognitive domains, including attention, memory, executive function, and processing speed. Notably, global measures WM microstructural abnormalities have been linked to poorer performance on tasks assessing sustained attention, vigilance, and working memory, reinforcing the relevance of diffuse structural changes in the cognitive dysfunction observed in RRMS (Alshehri et al., 2022; Vinciguerra et al., 2021). Increased MD and RD, particularly in regions affected by U-fiber lesions, were associated with reduced performance, suggesting that microstructural damage plays a pivotal role in disrupting cognitive processing.

Region-specific analyses provide additional insight into the neural substrates underlying cognitive dysfunction. The CC, a major commissural tract essential for interhemispheric communication, emerged as a consistent predictor of cognitive outcomes, with higher FA in the genu and splenium correlating with better outcomes in attentive subtests, processing speed and executive function (Pokryszko-Dragan et al., 2018; Bernabéu-Sanz et al., 2021). Similarly, thalamic microstructural changes, particularly in thalamic radiations, were strongly associated with cognitive flexibility (Bisecco et al., 2015; Wilting et al., 2016), pointing to the role of subcortical relay structures in MS-related cognitive deficits. The hippocampus, a critical hub for memory consolidation, also demonstrated significant DTI alterations, with increased MD correlating negatively with global cognitive performance (Gu et al., 2022). Functional disconnection within the hippocampus was also associated with working memory impairments (Tona et al., 2014), reaffirming its centrality in cognitive performance in MS subjects. Additionally, alterations involving the cerebellar peduncles, particularly the superior cerebellar peduncle, were associated with processing speed information, suggesting that cerebellar-cortical circuits contribute to cognitive efficiency beyond motor coordination (Elkhooly et al., 2023; Riccitelli et al., 2019). In addition to these key structures, the integrity of associative and projection fibers, including the cingulum, corona radiata, and inferior fronto-occipital fasciculus, was also linked to cognitive performance, notably in tasks involvingspeed and memory (Sbardella et al., 2013; Bernabéu-Sanz et al., 2021). In general, the integrity of interhemispheric connections and connecting structures is crucial; consequently, the presence of lesions and deterioration may lead to greater cognitive deficits related to MS. Further, these findings together support a network-based view of cognition in MS, in which distributed WM disruptions, rather than isolated lesions, may better explain the variability in cognitive decline. The use of DTI appears to be emerging as a potential standard in the management and monitoring of disease progression, as it can detect microstructural damage not visible through conventional MRI scans. These microstructural changes may evolve into more severe lesions or clinical manifestations. Therefore, early identification could facilitate timely interventions and the implementation of preventative strategies. In the case of RRMS, DTI may also anticipate the formation of active lesions or help identify the neuroanatomical basis of otherwise unexplained symptoms. WM damage disrupts brain networks and can have significant repercussions on cognitive functioning. Interestingly, even when no correlation is observed between DTI findings and cognitive performance, perhaps due to compensatory factors such as cognitive reserve, education level, disease duration, or age, these cases might still indicate areas of potential vulnerability. This provides an opportunity for early intervention and cognitive enhancement, before clinical deterioration becomes evident.

A combined and in-depth use of DTI alongside neuropsychological evaluation could improve the detection of disturbances not captured by conventional assessments. Furthermore, it could help explain subjective cognitive complaints or cognitive fatigue that are not attributable to other conditions but may stem from subtle WM alterations.

Several methodological limitations should be considered when interpreting the findings of this review. Primarily, the predominance of observational and cross-sectional studies among the DTI metrics and cognitive outcomes. Furthermore, considerable heterogeneity in MRI acquisition protocols (e.g., variations in field strength, diffusion encoding directions, and preprocessing pipelines) and in the selection and administration of cognitive assessment tools reduced the overall comparability and reproducibility of findings across studies. Such variability may affect both the sensitivity of diffusion measures and the strength of associations with cognitive performance. In addition, the potential influence of publication bias and selective reporting must be acknowledged, as studies reporting statistically significant or positive results are more likely to be published, thereby skewing the available evidence. Many studies also employed relatively small sample sizes, limiting statistical power and reducing the generalizability of their conclusions to broader populations. Given the substantial heterogeneity across studies, encompassing differences in study design, participant demographics, imaging methodologies (including the specific DTI metrics used), cognitive assessment approaches, and reported outcomes, a quantitative meta-analysis was not deemed appropriate. The lack of standardized reporting formats and consistent outcome measures further hindered the reliable aggregation of data. Moreover, variations in methodological rigor across the included studies may have introduced bias, further compromising the validity of potential pooled effect estimates. By explicitly addressing these sources of variability, we provide a more nuanced interpretation of the literature and contextualize differences observed across studies. Consequently, a qualitative synthesis was prioritized, thus enabling a more comprehensive andcontext-sensitive evaluation of the extant evidence base. This approach highlighted both converging findings and critical gaps in the literature. Therefore, in individuals with RRMS, WM integrity, assessed by DTI parameters, may represent a valuable biomarker for cognitive performance. Likewise, comprehensive cognitive assessments can provide insights into the specific brain regions associated with cognitive dysfunction. The connection between microstructural changes in network connectivity and key brain regions, such as global WM, hippocampus, cingulate, cerebral fascicles, and particularly interhemispheric WM, such as CC, thalamus, and cerebellar structure, and cognitive deficits underscores the link between structural connectivity and cognitive function. These findings highlight the potential of DTI as a valuable tool for assessing cognitive decline in MS and for guiding interventions aimed at preserving cognitive abilities in affected individuals.

## Data Availability

The original contributions presented in the study are included in the article/Supplementary material, further inquiries can be directed to the corresponding author.
