# Supplementary material for: Correlation of cognitive dysfunctions and diffusion tensor MRI measures in subjects with RRMS
Source: Front Aging Neurosci. 2025 Nov 13;17:1661821. doi: 10.3389/fnagi.2025.1661821 (PMC12657435; doi:10.3389/fnagi.2025.1661821)
Supplement: Supplementary file 1 [file Data_Sheet_1.docx]

**Supplementary Table 1: Overview of reported correlations between DTI metrics and cognitive tests in RRMS**

| **DTI Metric** | **Cognitive Test** | **Direction of Correlation** | **Number of Studies / Notes** |
| --- | --- | --- | --- |
| FA | SDMT | Positive | 28/34 studies (includes global WM, CC, thalamus, UF, cingulum, SLF) |
| MD | SDMT | Negative | 22/30 studies (global WM, CC, thalamus, cerebellum) |
| RD | SDMT | Negative | 15/20 studies (global WM, CC, UF, SLF) |
| FA | PASAT | Positive | 18/22 studies (CC, cingulum, SLF, corona radiata) |
| MD | PASAT | Negative | 12/18 studies (thalamus, CC, corona radiata) |
| RD | PASAT | Negative | 10/15 studies (CC, cingulum, SLF) |
| FA | tARCS | Positive | 1/1 study (global WM, Alshehri et al., 2022) |
| FA | BVMT / BVMT-R | Positive | 3/3 studies (DMN, hippocampus, CC) |
| MD | BVMT / BVMT-R | Negative | 2/2 studies (global WM, thalamus) |
| FA | VLMT | Positive | 2/2 studies (DMN, limbic network) |
| MD | VLMT | Negative | 1/1 study (global WM) |
| FA | MoCA | Positive | 2/2 studies (T1/T2 lesions, UFLs non-UFLs) |
| MD | MoCA | Negative | 2/2 studies (UFLs, T1/T2 lesions) |
| FA | HVLT-DR | Positive | 3/3 studies (CC, RD/FA associations) |
| RD | HVLT-DR | Negative | 2/2 studies (CC) |
| FA | 10/36 SRT | Positive | 3/3 studies (CC splenium, SLF, corona radiata) |
| MD | 10/36 SRT | Negative | 3/3 studies (CC splenium, thalamic radiation, corona radiata) |
| FA | WCST | Positive | 2/2 studies (CC, SLF) |
| MD | WCST | Negative | 2/2 studies (CC, SLF) |
| FA | MET / Hotel Task | Positive | 1/1 study (frontolateral WM) |
| ADC | MET / Hotel Task | Positive | 1/1 study (frontolateral WM) |
| FA | RAVLT | Positive | 2/2 studies (CC, cingulum) |
| FA | DET | Positive | 1/1 study (corticospinal tract, superior occipital-frontal fasciculus) |
| MD/RD/AD | DET | Negative | 1/1 study (corticospinal tract, superior occipital-frontal fasciculus) |

Legend: DTI: Diffusion Tensor Imaging, FA: Fractional Anisotropy, MD: Mean Diffusivity, RD: Radial Diffusivity, AD: axial diffusivities, ADC: Apparent Diffusion Coefficient, WM: White Matter, CC: Corpus Callosum, UF: Uncinate fasciculus, SLF: Superior Longitudinal Fasciculus, UFLs: U-fiber lesions, DMN: Default Mode Network, SDMT: Symbol Digit Modalities Test, PASAT: Paced Auditory Serial Addition Test, VLMT: Verbal Learning and Memory Test, tARCS: total Audio Recorded Cognitive Screen, BVMT: Brief Visuo-Motor Test-Revised, VLMT: Verbal Learning and Memory Test, MOCA: Montreal Cognitive Assessment, HVLT: Hopkins Verbal Learning Tests, 10/36 SRT: 10/36 Spatial Recall Test, WCST: Wisconsin Card Sorting Test, MET: Multiple Errands Test, RAVLT: Rey Auditory Verbal Learning Test, DET: Detection Task.
