# Supplementary material for: Correlation of cognitive dysfunctions and diffusion tensor MRI measures in subjects with RRMS
Source: Front Aging Neurosci. 2025 Nov 13;17:1661821. doi: 10.3389/fnagi.2025.1661821 (PMC12657435; doi:10.3389/fnagi.2025.1661821)
Supplement: Supplementary file 2 [file Data_Sheet_2.docx]

|  |  |  |  |  |  |  |
| --- | --- | --- | --- | --- | --- | --- |

| Supplementary Table2. Correlation between DTI Measures and Neuropsychological Test in RRMS |  |
| --- | --- |

| Author | DTI Measure | ROI | Tests of Neuropsicological Evalutation | Significant neuropsicological Correlation | Correlation coefficient | p-value |
| --- | --- | --- | --- | --- | --- | --- |
| Pokryszko-Dragan et al. (2018) | FA, ADC | Corpus Callosum, thalamus, cerebellum | PASAT SDMT (MSFC) | Corpus Callosum  FA (genu) ↑ SDMT  FA (splenium) ↑ SDMT  ADC (splenium) ↓ SDMT  Thalamus  ADC (right) ↓ SDMT  ADC (right) ↑ 9HPT  FA (left) ↓ 9HPT  Middle Cerebellar Peduncle (left)  ADC ↑ 9HPT | r = 0.33  r = 0.38  r = –0.29  r = –0.30  r = 0.38  r = –0.32  r = 0.31 | p = 0.022  p = 0.009  p = 0.046  p = 0.041  p = 0.008  p = 0.031  p = 0.032 |
| Abdulaziz Alshehri 2022 | FA, MD, RD,  AD | TBWM,  WML | tARCS  SDMT | **DTI ↔ Cognition Correlations**  FA/ tARCS ↑ FA/ Memory ↑ FA/ Fluency ↑  FA/ Attention ↑  RD/ tARCS ↓  RD/ Memory ↓ RD/ Fluency ↓  MD/ Memory ↓ MD/ Fluency ↓ | r = 0.41  r = 0.41  r = 0.35  r = 0.41  r = –0.43  r = –0.47  r = –0.45  r = –0.37  r = –0.39 | p ≤ .05  p ≤ .05  p ≤ .05  p ≤ .05  p ≤ .01  p ≤ .01  p ≤ .01  p ≤ .05  p ≤ .05 |
| Bisecco 2015 | DWI, FA,  MD | Thalamus, cortico- thalamic tracts,  cerebral volume | BRB-N SRT 10/36  SRT  SDMT PASAT WLG WCST | Increased in cortical regions  Frontal/ FA ↑  Motor/ FA ↑  Postcentral/ FA ↑  Occipital/ FA ↑  Atrophy and alterations in the cortico-thalamic tracts  Anterior thalamic regions ↑ Atrophy  Frontal tract ↑ MD  Motor tract ↑ MD  Postcentral tract ↑ MD  Temporal tract ↑ MD  Occipital tract ↑ MD  Temporal tract ↓ FA  Occipital tract ↓ FA |  | p = 0.02  p = 0.01  p = 0.005  p = 0.005  p = 0.009  p = 0.01  p = 0.002  p = 0.0003  p = 0.009  p = 0.007  p = 0.008 |
| Bernabéu-Sanz 2021 | FA,  MD | Brain Volumetry,  VBM,  TBSS | SDMT MAT global score MAT-orientation  k z-MAT-semantic  k z-MAT-free recall  k z-MAT-cued-recall  k z-MAT-episodic | Thalamic Volume ↑ SDMT  FA in Left Thalamus ↑ SDMT  MD in Thalamocortical Projections & SDMT  Frontal: L ↑  R ↑  Parietal: L ↑  R ↑  Temporal: L ↑  R ↑  WM Damage & Episodic Memory (M@T)  Left Uncinate Fasciculus ↑  Left Inferior Longitudinal Fasciculus ↑  Right Cingulum ↑ | r = 0.55  r = 0.56  r = 0.58  r = 0.55  r = 0.55  r = 0.57  r = 0.57  r = 0.477  r = 0.38 | p = 0.005  p = 0.03  p = 0.002  p = 0.002 p < 0.001  p = 0.001  p = 0.002  p = 0.001  p = 0.001  p = 0.009  p = 0.04 |
| Arzu Ceylan Has Silemek 2020 | FA/FOD, MD | Structural and functional connectivity for each one of seven subnetworks based on the Yeo atlas | TAP PASAT SDMT VLMT WMS BVMT RWT | Correlations with Structural Connectivity  SDMT ↔ Global Structural Graph Strength ↑ ,  BVMT Sum 1–3 ↔ Global Structural Graph Strength ↑  BVMT Recall ↔ Global Structural Graph Strength ↑ | r = 0.46  r = 0.55  r = 0.53 | p = 0.007  p = 0.001  p = 0.002 |
| Vinciguerra 2020 | FA, MD | Brain volumetry, TBSS,  Lesion volume | SDMT BRB-N SRT-LTS SRT-CLTR  SRT-D  SPART PASAT3 SPART-D  WLG | PSMD Correlations with Cognitive Tests  SDMT ↓  SRT-LTS ↓ (verbal memory)  SRT-CLTR ↓ (verbal memory)  SPART ↓ (visual memory)  WLG: ↑ (verbal fluency) | r = −0.70  r = −0.35  r = −0.37  r = −0.28  r = 0.25 | p < 0.001  p = 0.02  p = 0.016  p = 0.02  p = 0.04 |
| De Medeiros Rimkus 2011 | FA, MD,  AD, VOI | Corpus Callosum | BVMT WAIS III SDMT TMT STROOP FAS WCST categori association HVLT im. Rec. HVLT de.rec. ROCFde.re. | FA ↔ SDMT ↑  RD ↔ HVLT-delayed recall ↑  FA ↔ Logical Memory II ↑ | r = 0.46  r = 0.45  r = 0.41 | p = 0.02  p = 0.03  p = 0.05 |
| Dan Lou 2023 | DKI - MK, AK, RK, KFA | Brain Volumetry,  U- fiber. | MOCA DST SDMT | Kurtosis Fractional Anisotropy  Lesioned & Non-lesioned  KFA U-Fiber non Lesions ↑ DST  ↑ SDM    U-Fiber Lesions ↓ MoCA | r = 0.371  r = 0.399  r = −0.372 | p = 0.018  p = 0.010  p = 0.021 |
| Veréb 2022 | FA | TBSS | BICAMS  SDMT BVMT-R CVLT-II | DMN – Angular Gyrus ↑BVMT-R | r = –0.52, | *p* < 0.023 |
| Sbardella 2013 | FA, MD, AD, RD | Resting State Networks, WBVA,  TBSS | MSFC PASAT | FA ↑ PASAT2s | *r* = 0.47 | *p* = 0.005 |
| Sbardella 2015 | FA, MD, AD, RD | WBVA,  TBSS | PASAT MMSE MSFC | FA ↔ PASAT ↑  MD ↔ PASAT ↓  AD ↔ PASAT ↓  RD ↔ PASAT ↓ | n.r.  n.r.  n.r.  n.r. | p < 0.008  p < 0.008  p < 0.008  p < 0.008 |
| Sbardella 2016 | FA | Dentate functional connectivity | PASAT | FA ↔ PASAT↑ | n.r. | p < 0.005 |
| Mazerolle 2013 | FA, MD, AD, RD | TBSS | BDI-Fast Screen SDMT | FA (CC – body, genu) ↔ SDMT ↑  FA (PTR) ↔ ↑ SDMT  FA (PTR) ↔ ↑ SDMT  RD (PTR) ↔ ↓ SDMT  MD (PTR) ↔ ↓ SDMT  FA (CC – body, genu, splenium; UF; Corona Radiata) ↔ CTIP-SRT ↑  RD (corona radiata) ↔ CTIP-SRT ↓  MD (CC splenium) ↔ CTIP-SRT↓ | n.r.  n.r  n.r  n.r  n.r  n.r.  n.r.  n.r. | *p* < 0.01  *p* < 0.01  *p* < 0.01  *p* < 0.01  *p* < 0.01  *p* < 0.01  *p* < 0.01  *p* < 0.01 |
| Feiyue Yin 2023 | FA, MD | Deep gray matter,  Caudatete nucleus, putamen, globus pallidus, thalamus | MMSE  SDMT MOCA | **DGM – Microstructure (MD)**  Thalamus MD ↓ SDMT  Putamen MD ↑ EDSS  GP MD ↑ EDSS  **Disease Duration**  ↓GP volume  ↓ Thalamus volume  ↑ Thalamus MD | r = –0.419  r = –0.331  r = –0.478  r = –0.477  r = –0.406  r = 0.427 | p = 0.003  p = 0.023  p = 0.001  p = 0.001  p = 0.005  p = 0.003 |
| Tona 2014 | FA, MD, AD, RD | Thalamocortical functional connectivity | PASAT | **PASAT 3s** **↓ FC** with thalamus, cerebellum, cortical areas in all lobes  **PASAT 2s** **↓ FC** with above regions + cingulum + left hippocampus |  | *p < .05* |
| Fuqing Zhou 2016 | FA, MD, AD, RD | Thalamocortical system | PASAT | AD (prefrontal tract) ↔ ↓ PASAT | ρ = −0.606 | p = 0.013 |
| Riccitelli 2017 | FA, MD, AD, RD | Brain Volumetry,  WBWM | PASAT SDMT | SDMT ↔ ↑ Disease duration  SDMT ↔ ↑ EDSS  SDMT ↔ ↓ BPF  SDMT ↔ ↓ WMF  SDMT ↔ ↓GM atrophy in: • R anterior cingulate (BA24) • L postcentral gyrus (BA4) • R middle temporal gyrus (BA22)  **PASAT correlations**  PASAT ↔ ↓ BPF  PASAT ↔ ↓ GMF  PASAT ↔ ↓ WMF  PASAT ↔ ↓ WM damage in supratentorial tracts )  PASAT ↔ ↓GM atrophy in: • R thalamus • Caudate  • Putamen  • L pallidum  • R ACC (BA32) • Superior frontal gyrus (BA32/BA9;) • Precentral gyrus (BA6) • L STG (BA22) • R fusiform gyrus (BA19) | r = −0.20  r = −0.23  r = 0.21  r = 0.24  r = 0.23  r = 0.23  r = 0.22  r = 0.26  r = 0.16  r = 0.29  r = 0.10–0.44  r = 0.27  r = 0.37  r = 0.29  r = 0.31  r = 0.29  r = 0.26  r = 0.29  r = 0.28  r = 0.30 | p = .01  p = .002  p = .005  p < .002  *p < .001*  p < .0001  p = .03  p < .0001  *(p < .001)* |
| Savini 2019 | FA | 8 ROIs corresponding to the nodes of the resting state network and divided between right and left hemispheres: - L and R medial frontal cortex; -L and Rangular gyrus; -L and R precuneus/posterior cingulate cortex; -L and Rmiddle temporal gyrus | SDMT | **CIMS (RRMS subgroup)**  **SDMT ↑ FA-weighted GE** • GE(CBL-DMN) • GE(DMN)  • GE(CBL)  **CPMS**  SDMT score ↑ BPF | r = 0.87  r = 0.82  r = 0.80  r = 0.57 | p < .001  p < .001 p < .001  p < .001 |
| Hui Jing Yu et al. 2011 | FA, MD, AD, RD | WBVA,  WBWM,  NAWM | PASAT SDMT RAVLT | **SDMT ↓ ↔ FA** |  | *p < .01* |
| Wilting 2015 | FA, MD | WBVA,  WBWM,  Thalamus,  Basal Ganglia,  Frontal cortex | SDMT TAP PASAT VLMT dg5 VLMT dgt7 TMT-A TMT - B RWT | SDMT ↔ ↑ FA  SDMT ↔ ↓ MD  TMT‑B ↔ ↑ FA | r = +0.245  r = −0.308 = +0.308 | p = 0.032  p = 0.006  p = 0.007 |
| Kern 2014 | FA, AD, RD | Cingulus, Uncinate fasciculus, thalamo hippocampus | BDI PASAT SDMT BSRT WAIS 7/24 learning raw score  SRT | **Microstructural changes**  **FA ↓** in: • Cingulum  • UF  • Fornix |  | p = 0.008  p = 0.005  p = 0.0004 |
| Bozzali 2013 | ACM, FA | WBVA,  WBWM,  WBACM | MSFC BRB PASAT correct PASAT error | ACM (CC) ↔ PASAT ↓  ACM (R hippocampus) ↔ PASAT ↓  ACM (cerebellum) ↔ PASAT ↓ | n.r. | *p < 0.05* |
| Roca 2008 | FA, ADC | NABM in frontal lobe | PASAT(correct) MSFC MMSE ACE WAT.BA Vocabulary Subscale (WAIS)  Raven Colour Progressive Matrices  Paragraph Memory (immediate)-WMS  Paragraph Memory (long-term)-WMS  Recognition  Rey list Rey figure  Boston  F.A.S  Semantic Fluency  Token Test  Digits Forward  Digits Backward  TMT A  TMT B  Letters and Numbers WCST categories | PASAT ↔ ↑ FA (FL)  MET tasks achieved ↔ ↑ ADC (FL)  Hotel Task time deviation ↔ ↑ ADC | r = 0.64  r = 0.72  *r = 0.68* | p = 0.03  p = 0.01  *p = 0.02* |
| Elkhooly 2023 | FA, MD | Brain Volumetry,  TBWM | PASAT3 SDMT | Cognitive correlations  SDMT ↔ ↑ nGMV  PASAT‑3 ↔ ↑ nGMV (, )  White matter tract correlations (SDMT)  SDMT ↔ ↑ FA in: • L corticothalamic tract ,  • Superior cerebellar peduncle  • R medial lemniscus | r = 0.563  r = 0.369  r = 0.441  r = 0.512  r = 0.454 | p = 0.001  p = 0.05  p = 0.015  p = 0.004  p = 0.012 |
| Pardini 2013 | FA | Hippocampal volume, FA for the Uncinate Fasciculus and for the ventral division of the cingulum bundle | BRB-N SRT D 10/36 SRT 7/24 SPART | Memory correlations  Verbal memory ↔ ↑ Left NHV *,*  Spatial memory ↔ ↑ Right NHV | *r = 0.52*  *r = 0.56* | *p = 0.008*  *p = 0.004* |
| Preziosa 2016 | FA, MD | Brain Volumes,  WBWM, | BRB-N SRT 10/36SRT SDMT PASAT WLG WCST | **Specific cognitive deficits**  Attention & processing speed ↔ ↓ thalamic volume  Executive dysfunction ↔ ↓ IFG volume Visual memory impairment ↔ ↑ MD (splenium CC) | *r = 0.63*  *r = 0.65*  *r = −0.53* |  |
| Zhu 2022 | DKI-KFA, FA, MK, MD | Whole Brain Lesion Volume,  NAWM,  WBWM | MMSE MOCA SDMT | Cognitive correlations  Pure-T2Ls • MK ↔ ↑ MMSE  • FA ↔ ↑ MoCA  NAWM • KFA ↔ ↑ MMSE • FA ↔ ↑MMSE  • MK ↔ ↑MMSE  • MD ↔ ↑MMSE | *r = 0.331*  *r = 0.309*  *r = 0.360*  *r = 0.415*  *r = 0.369*  *r = 0.531* | *p = 0.024*  *p = 0.036*  *p = 0.014*  *p = 0.004*  *p = 0.012*  *p < .001* |
| Dineen 2012 | FA, AD, RD | Fornix regions, hippocampal, mammillary body and thalamic | Z score of: BVRT CVLTII CVLT-II recognition COWAT | Cognitive prediction  Visual recall performance predicted by: • Fornix FA *(R² = .31)* • Mammillary body volume |  | *p = .008*  *p = .038* |
| Sindhuja Tirumalai Govindarajan 2020 | FA, MD, AD, RD | Focal WM Lesions, WBWM,  Specific WM Tracts (Acoustic radiation  Callosal body, Cingulum  Corticospinal tract,  Fornix,  Inferior occipito-frontal fascicle,  Optic radiation,  Superior longitudinal fascicle,  Superior occipito-frontal fascicle,  Uncinate fascicle). | Z score of SDMT Cogstate brief battery: Detection (DET)  Identification (IDN) | Cognitive correlations  SDMT ↔ ↑ MD/RD/AD in corpus callosum & L corticospinal tract  DET score ↔ ↑ MD in R superior occipitofrontal fascicle |  | **p < .05** |
| Barone 2018 | FA | Corpus Callosum | MMSE BRB-N | FA → ↓ verbal fluency | n.r. | *p < .001* |
| X Lin et al. 2005 | ADC | Pyramidal tracts, Corpus Callosum | PASAT | **Structure–function correlations**  PASAT ↑ ↔ ADC (CC) *)*  Lesion burden associations ↔ PASAT | *r = 0.58*  *r = 0.50* | *p = 0.001*  *p = 0.0075* |
| X Lin 2007 | ADC | Corpus callosum | PASAT | PASAT correlations  PASAT ↑ ↔ CC MTR  PASAT ↓ ↔ CC ADC  PASAT ↑ ↔ CC area  PASAT ↓ ↔ total T2 lesion load | r = 0.47  r = −0.53  r = 0.42  r = −0.40 | p = 0.0046  p = 0.0012  p = 0.01  p = 0.017 |
| Xin-Quan Gu 2021 | FA, MD | WBWM,  Hippocampus | MOCA | Functional connectivity (FC)  MoCA ↓ ↔ FC (L hippocampus)  EDSS ↓ ↔ FC (R hippocampus) ( , )  Microstructural metric (MD)  MoCA ↓ ↔ MD (L hippocampus)  EDSS ↑ ↔ MD (L hippocampus) | r = −0.698  r =−0.649  r = −0.729  r = 0.653 | p = 0.025  p = 0.042  p = 0.017  p = 0.041 |
| Zhuowei Shi 2023 | KFA, FA, MK, MD | CELs,  non-CELs,  NAWM,  Perilesional WM | SDMT MMSE MOCA | Cognitive correlations  MoCA ↓ ↔ MD (L hippocampus)  SDMT ↑ ↔ KFA (perilesional WM of enhancing lesions) ,  DST ↑ ↔ KFA (IRLs-PLWM)  DST ↓↔ MD (IRLs-PLWM) | r = −0.518  r = 0.396  r = 0.44  r = −0.518 | p = .006  p = .041  p = .021  p = .006 |

**Legend of symbols:**

**↑ = increase / increment**

**↓ = decrease / increment**

**↔ = correlation**

**NS = not significant**

RRMS : Relapsing-Remitting Multiple Sclerosis; MS: Multiple Sclerosis; HC: Healthy Controls; WM: White Matter; GM: Gray Matter; FA: Fractional Anisotropy; MD: Mean Diffusivity; RD: Radial Diffusivity; AD: Axial Diffusivity; PSMD: Peak width of skeletonized mean diffusivity; KFA: Kurtosis Fractional Anisotropy; MK: Mean Kurtosis; nTBV: Normalized Total Brain Volume; nGMV: Normalized Gray Matter Volume; nWMV: Normalized White Matter Volume; NHV: Normalized Hippocampal Volume; BPF: Brain Parenchymal Fraction; WM: White Matter; WMF / GMF: White Matter Fraction / Gray Matter Fraction; TBWM: Total Brain White Matter; PASAT: Paced Auditory Serial Addition Test; SDMT: Symbol Digit Modalities Test; DST: Digit Span Test; MoCA: Montreal Cognitive Assessment; MMSE: Mini-Mental State Examination; MET: Multiple Errands Test; CBB: CogState Brief Battery; BSRT: Buschke Selective Reminding Test, DET / IDN: Detection / Identification tasks; DMN: Default Mode Network; CC: Corpus Callosum; SLF: Superior Longitudinal Fasciculus; ILF: Inferior Longitudinal Fasciculus; IFOF: Inferior Fronto-Occipital Fasciculus; PCR: Posterior Corona Radiata; VCB: Ventral Cingulum Bundle; UF: Uncinate Fasciculus; IRLs-PLWM: Perilesional White Matter of Iron Rim Lesions; ACM: Anatomical Connectivity Map; FC: Functional Connectivity; EDSS: Expanded Disability Status Scale; IFG: Inferior Frontal Gyrus; STG: Superior Temporal Gyrus; BA : Brodmann Area; TFCE: Threshold-Free Cluster Enhancement; L / R: left / right; FSS; Fatigue; HAMD: Hamilton Depression Scale
